# Dynamic Modelling of Mental Resilience in Young Adults: Protocol for a Longitudinal Observational Study (DynaM-OBS)

**DOI:** 10.2196/39817

**Published:** 2023-07-04

**Authors:** Carolin Wackerhagen, Ilya M Veer, Judith M C van Leeuwen, Zala Reppmann, Antje Riepenhausen, Sophie A Bögemann, Netali Mor, Lara M C Puhlmann, Aleksandra Uściƚko, Matthias Zerban, Julian Mituniewicz, Avigail Lerner, Kenneth S L Yuen, Göran Köber, Marta A Marciniak, Shakoor Pooseh, Jeroen Weermeijer, Alejandro Arias-Vásquez, Harald Binder, Walter de Raedt, Birgit Kleim, Inez Myin-Germeys, Karin Roelofs, Jens Timmer, Oliver Tüscher, Talma Hendler, Dorota Kobylińska, Erno J Hermans, Raffael Kalisch, Henrik Walter

**Affiliations:** 1 Research Division of Mind and Brain Department of Psychiatry and Psychotherapy, Charité Campus Mitte Charité - Universitätsmedizin Berlin, Corporate Member of Freie Universität Berlin, Humboldt-Universität zu Berlin, and Berlin Institute of Health Berlin Germany; 2 Department of Developmental Psychology University of Amsterdam Amsterdam Netherlands; 3 Donders Institute for Brain, Cognition, and Behaviour Radboud University Nijmegen Netherlands; 4 Faculty of Philosophy Berlin School of Mind and Brain Humboldt-Universität zu Berlin Berlin Germany; 5 Sagol Brain Institute Tel Aviv Sourasky Medical Center Tel Aviv Israel; 6 Sackler Faculty of Medicine Tel Aviv University Tel Aviv Israel; 7 Leibniz Institute for Resilience Research Mainz Germany; 8 Max Planck Institute for Human Cognitive and Brain Sciences Leipzig Germany; 9 Faculty of Psychology University of Warsaw Warsaw Poland; 10 Neuroimaging Center, Focus Program Translational Neuroscience Johannes Gutenberg University Medical Center Mainz Germany; 11 Sagol School of Neuroscience Tel Aviv University Tel Aviv Israel; 12 Institute of Medical Biometry and Statistics Faculty of Medicine and Medical Center University of Freiburg Freiburg Germany; 13 Freiburg Center for Data Analysis and Modelling University of Freiburg Freiburg Germany; 14 Division of Experimental Psychopathology and Psychotherapy Department of Psychology University of Zurich Zurich Switzerland; 15 Department of Psychiatry, Psychotherapy and Psychosomatics Psychiatric University Hospital University of Zurich Zurich Switzerland; 16 Center for Contextual Psychiatry Department of Neurosciences Katholieke Universiteit Leuven Leuven Belgium; 17 Stichting imec Nederland Holst Centre Eindhoven Netherlands; 18 Behavioural Science Institute Radboud University Nijmegen Netherlands; 19 Institute of Physics University of Freiburg Freiburg Germany; 20 Centre for Biological Signalling Studies and Centre for Integrative Biological Signalling Studies University of Freiburg Freiburg Germany; 21 Department of Psychiatry and Psychotherapy Johannes Gutenberg University Medical Center Mainz Germany; 22 School of Psychological Science Tel Aviv University Tel Aviv Israel

**Keywords:** resilience, stress, stressor reactivity, resilience factors, mental health, stress disorders, longitudinal, prospective, neuroimaging, ecological momentary assessment, mobile phone

## Abstract

**Background:**

Stress-related mental disorders are highly prevalent and pose a substantial burden on individuals and society. Improving strategies for the prevention and treatment of mental disorders requires a better understanding of their risk and resilience factors. This multicenter study aims to contribute to this endeavor by investigating psychological resilience in healthy but susceptible young adults over 9 months. Resilience is conceptualized in this study as the maintenance of mental health or quick recovery from mental health perturbations upon exposure to stressors, assessed longitudinally via frequent monitoring of stressors and mental health.

**Objective:**

This study aims to investigate the factors predicting mental resilience and adaptive processes and mechanisms contributing to mental resilience and to provide a methodological and evidence-based framework for later intervention studies.

**Methods:**

In a multicenter setting, across 5 research sites, a sample with a total target size of 250 young male and female adults was assessed longitudinally over 9 months. Participants were included if they reported at least 3 past stressful life events and an elevated level of (internalizing) mental health problems but were not presently affected by any mental disorder other than mild depression. At baseline, sociodemographic, psychological, neuropsychological, structural, and functional brain imaging; salivary cortisol and α-amylase levels; and cardiovascular data were acquired. In a 6-month longitudinal phase 1, stressor exposure, mental health problems, and perceived positive appraisal were monitored biweekly in a web-based environment, while ecological momentary assessments and ecological physiological assessments took place once per month for 1 week, using mobile phones and wristbands. In a subsequent 3-month longitudinal phase 2, web-based monitoring was reduced to once a month, and psychological resilience and risk factors were assessed again at the end of the 9-month period. In addition, samples for genetic, epigenetic, and microbiome analyses were collected at baseline and at months 3 and 6. As an approximation of resilience, an individual stressor reactivity score will be calculated. Using regularized regression methods, network modeling, ordinary differential equations, landmarking methods, and neural net–based methods for imputation and dimension reduction, we will identify the predictors and mechanisms of stressor reactivity and thus be able to identify resilience factors and mechanisms that facilitate adaptation to stressors.

**Results:**

Participant inclusion began in October 2020, and data acquisition was completed in June 2022. A total of 249 participants were assessed at baseline, 209 finished longitudinal phase 1, and 153 finished longitudinal phase 2.

**Conclusions:**

The Dynamic Modelling of Resilience–Observational Study provides a methodological framework and data set to identify predictors and mechanisms of mental resilience, which are intended to serve as an empirical foundation for future intervention studies.

**International Registered Report Identifier (IRRID):**

DERR1-10.2196/39817

## Introduction

### Background

Stress-related disorders, such as depressive disorders, anxiety disorders, posttraumatic stress disorder, and addiction, are highly prevalent globally and pose a significant burden on individuals, the economy, and society in general [1-4]. Years lost to disability owing to mental disorders have increased by 45.2% from 1990 to 2016 [3], and mental disorders are associated with a reduced life expectancy by a median of 10.1 years [5]. Mental and substance use disorders are particularly prevalent in people in their 20s, and anxiety disorders and major depression have a high long-term stability [6]. Many mental health studies have focused on the mechanisms and predictors of vulnerability (risk) and dysfunction. However, we and others have argued that a focus on resilience, aiming to investigate factors and mechanisms contributing to the maintenance and recovery of mental health despite adversity [7], is a complimentary, helpful approach to reveal novel intervention targets and to help avert (chronic) mental health problems before they develop. Here, we describe the protocol of a longitudinal study designed to gain a better understanding of resilience.

Resilience is the maintenance of, or quick recovery toward, mental health during and after times of adversity, such as trauma, difficult life circumstances, challenging life transitions, or physical illness [8,9]. It is becoming increasingly clear that resilience is the result of a dynamic process of successful adaptation to stressors [9-14]. Besides person-environment interactions and the activation of dispositional coping strategies, there is accumulating evidence that individuals *change* while they successfully cope with stressors. These adjustments can manifest at various levels, such as altered perspectives on life [15], the emergence of new strengths or competencies [16], partial immunization against the effects of future stressors [17-19], or epigenetic alterations and modified gene expression patterns [20,21]. Neurobiological studies in animal models indicate that adjustments at the brain level are causal for the preservation of normal behavior [22-25]. From a dynamic perspective, resilience results from change and not just inertia, insensitivity to stressors, or merely a passive response to adversity.

Consequentially, resilience should no longer be understood as a fixed personality trait or predisposition (the *resilient personality*) that will determine successful coping independent of other factors. Rather, we should take a dynamic stance and investigate the complex, interactive, and time-varying processes (*resilience processes*) that lead to a positive long-term outcome relative to the number of stressors to which an organism is exposed. These processes will partly be determined by individual dispositions, including traits, habits and skills, beliefs, genotype, brain architecture, and physical constitution (“resilience factors”). Although some of those will be quite stable and define a person’s typical coping patterns throughout a stressful life period, other resilience factors may themselves undergo change, for instance, by increasing their effectiveness or frequency of use. The latter would constitute allostatic resilience processes, in which the system learns to change its mode of operation to remain stable. Allostatic adjustment is more likely when an external perturbation (a stressor) taxes the system. In contrast, homeostatic resilience processes are defined as mental stability in the absence of individual change [13].

By definition, resilience as an outcome cannot be measured through any one-time (cross-sectional) assessment (eg, a questionnaire, brain scan, and genotype) performed before adversity occurs, as the trait-like conceptualization of resilience implies. Instead, resilience can only be determined by assessing both stressors and mental health longitudinally, thus capturing the dynamic nature and time course of the stressors, as well as the changes in mental health that these stressors may or may not induce [9,11]. This should be complemented by assessing potential resilience factors at study baseline and, ideally, also repeatedly during the course of observation [13]. Moreover, the factors and processes of resilience should ideally be studied at different levels of organization, from the molecular (including genetic), physiological, neural, and cognitive levels to behavioral, experiential, and social levels [26].

### This Study

The observational study Dynamic Modelling of Resilience–Observational Study (DynaM-OBS) of the Consortium “DynaMORE (Dynamic Modelling of Resilience),” funded by the European Union Horizon 2020, is designed to unravel such multilevel mechanisms of outcome-based resilience. The targeted sample is young students who find themselves in the transition from family and school life to work or academics—a life period characterized by new unfamiliar environments and demands, which is associated in some individuals with the exacerbation of existing, or the onset of new, stress-related psychological problems. The rationale of focusing on young people is that many mental disorders have their first onset or even peak during this critical life transition phase [27], and stress-related mental problems appear to be a particular problem in the student population [28-33]. To enrich our sample with at-risk individuals, we added two further inclusion criteria: participants must (1) have a history of at least 3 adverse life events [34] and (2) score in the mid-to-high range in the General Health Questionnaire (GHQ), a self-report instrument for internalizing symptoms [35], in addition to being a student or undergoing an apprenticeship. The study started in October 2020, when the second wave of the COVID-19 pandemic began in Europe, which formed an additional general stressor to the sample population at the time of data assessment [36].

DynaM-OBS is a longitudinal study with a baseline assessment, a dense observation period of 6 months (longitudinal phase 1), and a subsequent less dense observation period of 3 months (longitudinal phase 2) for each participant. DynaM-OBS features four core components: (1) an extensive baseline characterization (baseline battery) measuring potential social, psychological, and biological resilience factors; (2) a biweekly web-based assessment of stressor exposure and mental health during phase 1; (3) repeated measurement of a subset of potential resilience factors at different time points during the study; and (4) every 4 weeks, 1-week–long ecological momentary assessments (EMAs) and ecological physiological assessments (EPAs) of mood and stress reaction patterns during real life conducted using smartphones and wristbands, respectively.

The DynaM-OBS biweekly stressor and mental health assessments (study core component 2) apply the frequent stressor and mental health monitoring (FRESHMO) paradigm introduced recently to operationalize and measure resilience in longitudinal studies [13]. In the data analysis, we will use normative modeling of stressor reactivity (SR) as an approximation of resilience. Specifically, we will regress participants’ average mental health problem score from all assessment time points in phase 1 over their average stressor exposure score from the same time points, and thus, we will establish the sample’s normative stressor exposure–mental health problem relationship during the study. At any single assessment time point or series of subsequent time points, the deviation of a participant’s (average) mental health problem score from the norm relationship (its regression residual) expresses the participant’s individual mental health reactivity to stressor exposure during that time window. A positive SR score reflects higher than predicted reactivity, whereas a negative SR score reflects lower than predicted reactivity. Importantly, SR scores inherently correct for different degrees of stressor exposure between individuals and can thus be compared between them.

On this basis, the inverse of the SR score calculated from the average stressor exposure and mental health problem scores over the entire longitudinal phase 1 time window of the study can be considered a dimensional measure of a participant’s outcome-based resilience over these 6 months. We will also build within-participant time courses of SR scores by building the score in sliding windows of several averaged biweekly stressor exposure and mental health problem measurements. This permits us to index temporal fluctuations in reactivity and to detect potential substantial changes that would be a strong indicator of allostatic adjustment at the level of resilience factors, without which relevant increases or decreases in reactivity are difficult to imagine.

Thus, the time series–based approach to SR assessment goes beyond the mere prediction of a longer-term resilience outcome (here, the inverted 6-month SR score of phase 1) from a single measurement of more or less stable resilience factors (here, DynaM-OBS baseline battery, study core component 1). In combination with repeated measurement of resilience factors (DynaM-OBS core component 3)—some biweekly and some at study entry and study exit—the approach allows for relating changes in the outcome to potentially underlying allostatic changes in resilience factors (toward the good or bad). Another more distal source of change in the outcome may lie in the stressor exposure itself, which may increase to a degree that the system reacts allostatically by ideally strengthening the existing or developing new coping mechanisms, which then, in turn, improve SR [13].

Core component 4 of DynaM-OBS (EMA and EPA) serves to examine whether stressor exposure, mental health, and SR measured using web-based questionnaires over periods of weeks or months (refer to component 2) are reflected in individuals’ patterns of SR measured at a higher temporal frequency in real life. EMA combined with EPA will allow us to quantify, among others, the frequency of self-reported real-life stressors, the magnitude of mood or physiological changes in response to these stressors, the speed of recovery, and the alterations of recovery speed.

### Relation to Other Studies

The FRESHMO paradigm [13] in its combination with repeated resilience factor measurement is or has been used in several other longitudinal studies by our consortium and partners, including the studies “MARP (Mainz Resilience Project)” (study protocol in preparation, functional magnetic resonance imaging [fMRI] battery and results published by Kampa et al [37,38]), “LORA (Longitudinal Resilience Assessment)” [39], and “DynaCORE-L (The DynaMORE Longitudinal Study on Psychological Resilience to the Mental Health Consequences of the Corona Crisis)” [40]. MARP and LORA are ongoing since 2016 and are monitoring stressors and mental health every 3 months and resilience factors approximately every 1.5 years over many years of participants’ lives (refer to the publication by Kalisch et al [13]). DynaCORE-L was conducted over 6 weeks during the early phase of the COVID-19 pandemic in 2020 and involved weekly stressor, health, and resilience factor monitoring. Similar to DynaM-OBS, all studies also include baseline resilience factor assessment. DynaM-OBS thus covers a time range lying in between MARP and LORA on the one hand and DynaCORE-L on the other hand. A similar approach is also used in the ongoing Healthy Brain Study, which uses 3 waves of assessments to calculate dynamic resilience scores [41].

In DynaCORE-L, we observed that several baseline resilience factors prospectively predicted average SR over the full monitoring period, whereas week-to-week changes in resilience factors were not predictive of SR changes in the subsequent week [42]. This indicates that allostatic adaptation processes may occur over longer timescales. Another possibility is that the relatively mild stressor exposure in DynaCORE-L, which occurred after the first wave of the pandemic had subsided, permitted homeostatic coping. Owing to its longer time range and more severe at-risk constellation, DynaM-OBS should be more sensitive than DynaCORE-L in detecting allostatic processes. At the same time, DynaM-OBS allows us to test the generalizability of the baseline resilience factors discovered in DynaCORE-L to a different sample. Another source of hypotheses is the unpublished preliminary analyses of the MARP and LORA data sets. In synopsis with the anticipated results from these long-term studies, DynaCORE-L and DynaM-OBS will yield a good picture of the temporal dynamics of allostatic resilience processes.

DynaCORE-L had further detected that week-to-week changes in stressor exposure predate subsequent changes, specifically in the resilience factor of active behavioral coping, which were, however, not translated into improvements in SR. Again, this may have been the consequence of the limited duration or limited exposure severity in the study. With DynaM-OBS we, therefore, also aim to detect mediated relationships between stressors, resilience factors, and SR. The addition of EMA and EPA is intended to detect potential relationships at much higher (within-week and within-day) temporal resolution, which is possible neither in DynaCORE-L nor in MARP and LORA.

In addition to answering questions about resilience factors and processes, a purpose of DynaM-OBS is to establish a methodological framework for a subsequently planned intervention study (DynaMORE intervention study [DynaM-INT]) that aims to find new ways to prevent stress-related mental illness in at-risk individuals with the help of mobile training apps targeting specific resilience factors. In the context of such interventions, regular monitoring of SR and resilience factors can provide information on the desired outcome (lastingly reduced SR) and potential working mechanisms (lastingly strengthened resilience factors), whereas a baseline battery of both resilience and risk factors (RFs) may inform us about the individual characteristics that predict intervention success.

### Statistical Methods Development

The conceptual framework and the types of data generated by DynaM-OBS require new analysis methods. Although the time-sensitive regression models used in DynaCORE-L were appropriate for analyzing time point–to–time point (week-to-week) effects, they were also limited. Notably, they only tested each of the multiple, partially correlated resilience factors in a separate model, and they did not consider interrelations between resilience factors; they did not test for time-lagged effects between either stressors and resilience factors or resilience factors and SR extending beyond 1 week; and they did not test for changes or change points in the time series of stressor exposure, resilience factors, or SR. Therefore, we also use DynaM-OBS (together with the other mentioned studies) as a test bed for new methodological developments. These include regularized regression methods [36,43], network models [12], landmarking methods [13], and individualized deep dynamic methods in combination with deep generative models, allowing, for example, additional quantification of resilience, multiple imputation, and dimension reduction [43]. We will also use the data for potential improvements in the calculation of SR scores, including partial least squares methods [44], serving notably to improve the mental health problem variance explanation by stressor exposure and the better integration of stressors of various sources into stressor exposure [13]. Thus, DynaM-OBS has a strong exploratory character from a methodological perspective.

### Research Questions and Hypotheses

Nevertheless, we formulate a set of hypotheses that will be tested using established methods, as described in the *Methods* section. A major theoretical background of DynaM-OBS is Positive Appraisal Style Theory of Resilience (PASTOR) [9,45]. Positive appraisal style (PAS) is the tendency to appraise potential threats to one’s goals and needs (stressors) in a way that avoids unnecessarily negative but also highly unrealistically positive (delusional) appraisals. Instead, appraisals typically produced by individuals exhibiting PAS range from realistic to slightly unrealistically positive appraisals. Therefore, positive appraisers generate appropriate, optimally regulated stress reactions that are sufficient to cope with a threat but do not use more resources than necessary and have more time for recovery and rebuilding of resources and more opportunity to make growth experiences than individuals who are inclined toward catastrophizing, pessimism, or helplessness.

### Questionnaires

DynaM-OBS uses a PAS self-report questionnaire developed to assess the cognitive processes or mental operations that lead to positive appraisal contents (Perceived Positive Appraisal Style Scale—process focused [PASS-process]; refer to the *Methods* section) in its baseline battery, its biweekly web-based monitoring, and in the exit questionnaire battery applied in month 9 of the study. We hypothesize (a) that the baseline PASS-process negatively predicts the SR score covering the first 6 months of the study (phase 1, henceforth: SR_1_), (b) that changes in PASS-process from time point to time point in the biweekly monitoring inversely cofluctuate with the corresponding SR score, (c) that such changes also negatively predict time-lagged changes in SR (where the duration of the PASS-process change necessary to entail SR changes and the duration of the time lag are to be explored), and (d) that changes in PASS-process from study baseline to study exit are negatively related to the SR score covering the entire study period (phases 1 and 2, SR_1+2_).

An alternative PAS self-report instrument used in the DynaM-OBS baseline and exit batteries focuses on positive appraisal content instead of focusing on the processes that generate such appraisals (Perceived Positive Appraisal Style Scale–content focused [PASS-content]; refer to the *Methods* section). Hypotheses a and d, therefore, analogously apply to PASS-content.

In this manner, we will also test other baseline and exit questionnaires that specifically assess positive appraisal tendencies on single threat appraisal dimensions, namely, the dimensions of threat probability (optimism), threat coping potential (general self-efficacy and control), and threat magnitude and costs (inverted anxiety sensitivity score).

In the combined multivariate analysis using regularized regression of the PAS scales and the latter instruments, we will also address the question of which of these scores are best in explaining SR. In a complementary approach using factor analysis, we will ask whether there are interpretable latent variables across questionnaires that can be considered dimension-reduced representations of the PAS construct and whether these predict SR.

Finally, we will ask whether PAS (assessed with the “winning” instrument or instruments in the comparative multivariate analysis or, alternatively, with suitable cross-questionnaire factors) mediates the effects of perceived social support on SR and whether its effect on SR is in turn mediated by perceived good stress recovery (refer to the studies by Kalisch et al [9] and Veer et al [36] for rationale). For these mediation analyses, we will again use either baseline scores (for explanation of SR_1_; hypothesis a) or baseline-to-exit change scores (for explanation of SR_1+2_; hypothesis d).

The complete battery of RFs assessed in the baseline and exit questionnaires will be evaluated for its ability to explain SR scores in a more exploratory manner, using the abovementioned and potential newly developed methodological approaches.

### Neuroimaging

In the development of PASTOR, we have emphasized the problems associated with self-report assessment in general (which knows various sources of bias) and self-report assessment of appraisal processes and contents in particular (not all of which may be accessible to consciousness and be verbally reportable) [9]. Therefore, we also use a complementary approach, namely, to indirectly index the effectiveness and efficiency of participants’ positive appraisal processes through objective measures of their behavioral, physiological, or neural reactions to stressors in the laboratory or in real life. To this end, we generated a baseline neuroimaging battery where fMRI tasks are (partly) accompanied by behavioral and physiological recordings. The battery includes tasks from the corresponding MARP battery (reward sensitivity, differential fear conditioning, and situation-focused volitional reappraisal [37]), all of which provide reproducible imaging data [38] and were either found to be predictive of SR in initial analyses in MARP (unpublished) or were retained because of their special theoretical interest (refer to the study by Kampa et al [37] for task rationales in the context of PASTOR). A purely behavioral stress reactivity and recovery task in the MARP battery is replaced in the DynaM-OBS battery using a dedicated neuroimaging variant [46,47]. To link the battery with other neuroimaging cohort studies, a frequently used implicit emotional processing task is also used [48,49]. These tasks are complemented by structural and resting-state fMRI measurements.

For the neuroimaging data, we hypothesize (based on initial MARP findings and PASTOR) that SR_1_ will be negatively predicted by activity related to (a) high gain anticipation (gain>zero anticipation contrast), (b) low loss anticipation (loss>zero anticipation contrast), (c) high threat safety discrimination during the processing of fear-conditioned stimuli (CS) (threat-predictive CS+ > non–threat-predictive CS- contrast), (d) low threat generalization (CS- contrast), (e) high volitional reappraisal (R>NR contrast). Finally (f), we hypothesize that SR_1_ will inversely correlate with amygdala-ventromedial prefrontal functional connectivity during implicit emotion processing as measured using a faces versus shapes matching paradigm [50]. For details on contrasts, refer to the studies by Kampa et al [37] and Wackerhagen et al [50]. Similar to the questionnaire measures, PASTOR-related neuroimaging measures will be additionally analyzed using multivariate regression and factor analysis to identify best or latent predictors, respectively, of SR. We will also attempt cross-modality analyses including both questionnaires and neuroimaging measures.

Similar to longitudinal resilience data, the neuroimaging data will be used for the development of analysis methods.

### Other Data Modalities and Hypotheses

PASTOR claims that the effects of other social, psychological, and biological resilience factors on resilience are mediated by how they shape PAS [9]. In this framework, we used a range of additional questionnaires, neuropsychological tests, and biological samples (blood for genotyping, DNA methylation, plasma proteome analysis, and also cytokines at 1 site; stool for gut microbiome analysis; and saliva for cortisol analysis). Well-known risk factors and sociodemographic variables were also assessed. The latter will be included as covariates in several planned analyses, along with other potentially confounding factors such as the study site.

## Methods

### Study Centers and Study Period

The data were acquired in a multicenter setting at 5 research sites: Charité–Universitätsmedizin Berlin, Department of Psychiatry and Psychotherapy in Berlin, Germany; Universitätsmedizin Mainz, Neuroimaging Center (NIC) in Mainz, Germany; Donders Centre for Cognitive Neuroimaging (DCCN) in Nijmegen, The Netherlands; Sagol Brain Institute, Tel Aviv University (TAU) and Tel Aviv Soursaky Medical Center, Tel Aviv, Israel; and University of Warsaw, Faculty of Psychology in Warsaw, Poland. Each site’s official language was used to communicate with the participants, as well as in the study materials (informed consent form, tasks, questionnaires). Participants enrolled in the study between October 2020 and September 2021, which was during the second wave of the COVID-19 pandemic, as indicated in the details provided in Multimedia Appendix 1. Phase 1 assessments were completed in March 2022, and phase 2 assessments were completed in June 2022.

### Participants

A total of 250 mentally healthy male or female participants who were studying or in vocational training at the time of recruitment were planned to be included at the 5 research sites (n=50 each). The exact number of participants per site will be reported in the follow-up reports. The permitted age range was 18-25 years at all sites, except for TAU, where the age range was 18-27 years because most young adults in Israel complete 2 to 3 years of military service and spend 1 year abroad before entering vocational training or university. Participants were included if they had experienced ≥3 stressful life events [34], which they rated as burdening, and if they reported an elevated level of general psychopathology (internalizing symptoms), defined by a score of >20 in the GHQ-28 [35]. The complete list of the inclusion criteria is provided in Table 1.

**Table 1:** List of inclusion criteria and format in which they were assessed. Participants who were found eligible for criteria 1 to 10 in the anonymous web-based screening were invited to an on-site interview to check eligibility for criteria 9 to 13, to receive written and verbal information about the study, and to provide written informed consent.

Number	Criterion	Format
1	Age between 18 and 25 years (18 and 27 years at Tel Aviv University)	Web-based
2	≥3 life events rated as burdening	Web-based
3	GHQ-28^a^ score of ≥20	Web-based
4	BMI between 18 and 27	Web-based
5	No hormonal treatment and no consumption of steroids or treatment with steroids	Web-based
6	Proficiency in the official language of the country of study enrollment (minimum level of C1 in the Common European Framework of Reference for Languages)	Web-based
7	Eligibility to participate in ecological physiological assessment using a wearable device (no skin disease in the wrist or chest area, no medical condition that increases the risk of infection through electrodes, and no medication with phototoxic side effects)	Web-based
8	Eligibility to participate in the fear conditioning task (no skin allergy or allergy to adhesive electrodes)	Web-based
9	No lifetime diagnosis of any severe mental or organic disorder that affects neurodevelopment owing to its pathological mechanism or treatment (eg, schizophrenia, bipolar disorder, anorexia or bulimia nervosa, attention-deficit hyperactivity disorder, autism spectrum disorder, meningitis, epilepsy, multiple sclerosis, stroke, brain cancer, brain concussion, or coma)	Web-based and interview
10	Eligibility for undergoing the functional magnetic resonance imaging protocol (normal or corrected-to-normal eyesight, no hearing impairment, no claustrophobia, no nonremovable ferromagnetic metal in or on the body, not pregnant, and no large tattoo on the head or neck area)	Web-based and interview
11	No diagnosis within 9 months before inclusion of any mental disorder other than a mild depressive episode (ICD-10^b^ F32.1), tobacco abuse or dependence (ICD F12), or substance abuse, as assessed by trained psychologists using the Mini-International Neuropsychiatric Interview [51]	Interview
12	No consumption of any psychoactive drug or substance up to 4 weeks before the first psychological assessment and before the magnetic resonance imaging assessment	Interview
13	The participant has received all relevant information about the study, is able to obtain full insight and is fully contractually capable, is willing and able to comply with the protocol, and agrees to participate by giving written consent	Interview

^a^GHQ-28: General Health Questionnaire.

^b^ICD-10: International Classification of Diseases, Version 10.

### Ethics Approval and Consent to Participate

The study was reviewed and approved by the local ethics committees of all participating sites: the ethics committee of Charité–Universitätsmedizin Berlin, Germany (ethics approval number [EAN] EA1/319/19); the ethics committee of Radboud University Medical Center, Nijmegen, The Netherlands (Commissie Mensgebonden Onderzoek Arnhem-Nijmegen; EAN 2019-5613); the ethics committee of TAU, Tel Aviv, Israel, and the Helsinki committee of Tel Aviv Souraski Medical Center (EAN 0055-19-TLV); the ethics committee of the State Medical Board of Rhineland-Palatinate, Mainz, Germany (EAN 2019-14731_1); and the Ethics Committee for Scientific Research at Faculty of Psychology, University of Warsaw (Komisja Etyki Badań Naukowych Wydziału Psychologii Uniwersytetu Warszawskiego; EAN 03/04/2020), Warsaw, Poland. All study participants provided written informed consent.

### Materials

#### Self-Report Variables

Self-report variables included demographic characteristics, stressor exposure, mental health, potential psychological resilience, and, to a lesser extent, risk factors. RFs are grouped into primary and secondary RFs. Primary RFs are of main interest in this study based on previous findings and the theoretical background of our consortium [9,36,40], whereas secondary RFs are based on hypotheses drawn from the literature. Where available, validated versions of the questionnaires and their translations into the site-specific languages were used. Self-developed questionnaires are provided at the Center for Open Science, Open Science Framework (OSF) [52]. Refer to Tables 2-5 for an overview of all questionnaires and Table S1 in Multimedia Appendix 2 [37,53-55] for an overview of all collected sociodemographic information. Refer to Multimedia Appendix 3 for an overview of validation studies of all questionnaires and their translated versions used at the different study sites.

**Table 2:** List of self-report questionnaires on stressor exposure. Please note that only the original publications are cited here and not the validation studies of versions translated into the 4 study languages. Self-developed questionnaires are provided at the Center for Open Science [52].

Name	Construct
Life Events Questionnaire	A total of 28 stressful life events (eg, death of a friend or family member, separation or divorce of the parents, and illness or injury). For each event, participants indicate whether and at what age it has occurred and how positive or burdensome it has been experienced [34].
List of COVID-19–related stressors	A list of 23 stressors specific to the COVID-19 pandemic (eg, being at increased risk for an infection, loss of social contact, and having COVID-19 symptoms), for which participants report whether the situation occurred and how burdensome it was perceived on a 5-point scale. The list was self-developed in March 2020 for the DynaCORE^a^ studies on psychological resilience during the COVID-19 pandemic [36,40].
Mainz Inventory of Microstressors	A total of 58 minor stressors of daily life (eg, loss or displacement of an object, conflict, bad weather, and traffic). Participants report whether the events have occurred and how straining they were experienced on a 5-point scale [56].

^a^DynaCORE: DynaMORE Study on Psychological Resilience to the Mental Health Consequences of the Corona Crisis.

**Table 3:** List of self-report questionnaires on mental health status. Please note that only the original publications are cited here and not the validation studies of versions translated into the 4 study languages.

Name	Construct
General Health Questionnaire	Symptoms of anxiety, depression, insomnia, social problems, and somatic symptoms. This inventory is designed to capture the inability to carry out normal functions and the appearance of new and distressing phenomena in the general population (28 items) [35].
Revised Symptom Checklist 90	Psychological distress in terms of 9 primary symptom dimensions including somatization, obsessive-compulsive, interpersonal sensitivity, depression, anxiety, hostility, phobic anxiety, paranoid ideation, and psychoticism (90 items) [57].
WHO^a^ Disability Assessment Schedule	Functioning and disability in accordance with the International Classification of Functioning, Disability, and Health (12 items) [58].

^a^WHO: World Health Organization.

**Table 4:** List of self-report questionnaires used to assess primary resilience and risk factors. Please note that only the original publications are cited here and not the validation studies of versions translated into the 4 study languages. Self-developed questionnaires are provided on the Open Science Framework website [52].

Name	Construct
Brief Resilience Scale	The subjective ability to cope with and recover from stress (10 items) [59].
Cognitive Emotion Regulation Questionnaire	Different strategies of emotion regulation such as self-blame, other blame, rumination, catastrophizing, positive refocusing, planning, positive reappraisal, putting into perspective, and acceptance (18 items) [60].
Coping Orientation to Problems Experienced Inventory	Emotion regulation strategies such as self-distraction, active coping, denial, substance use, use of emotional support, use of instrumental support, behavioral disengagement, venting, positive reframing, planning, humor, acceptance, religion, and self-blame (28 items) [61].
General Positive Appraisal Style Scale–content based	General style of positive appraisal in stressful situations, focusing on appraisal contents (29 items; self-developed). Validation and condensation into a PASS-content^a^ in progress.
General Self-Efficacy Scale	Perceived ability to cope with a variety of difficult demands in life (10 items) [62].
Internal External Locus of Control-4	Degree to which individuals perceive themselves the outcomes of their behavior to be determined by their own actions or by forces outside their control (4 items) [63].
Life Orientation Test–Revised	Dispositional optimism and pessimism (10 items) [64].
NEO^b^-Neuroticism	Neuroticism scale of the NEO Five Factor Inventory (12 items) [65].
Oslo 3 Item Social Support Scale	Degree to which participants perceive themselves as surrounded by people who are close, concerned, and supportive [66].
Perceived Positive Appraisal Style Scale—process based	Assessment of positive appraisal style focusing on cognitive processes that generate positive appraisal contents in stressful situations (as opposed to assessment of the resulting appraisal contents themselves, as in PASS-content above in this section). PASS-process^c^ includes correlated items of the Brief COPE^d^, the CERQ^e^ short, as well as 2 own-formulated items on distancing (detachment), which are all readily interpretable as indexing processes leading to positive appraisals (distancing, positive reappraisal, acceptance, putting into perspective, and humor). A previous version is referred to as PASS^f^ in the study by Veer et al [36].
Psychological Flexibility Questionnaire	Subjective psychological flexibility, assessed via 5 factors including positive perception of change, characterization of the self as flexible, self-characterization as open and innovative, a perception of reality as dynamic and changing, and a perception of reality as multifaceted (20 items) [67].

^a^PASS-content: Perceived Positive Appraisal Style Scale—content focused.

^b^NEO: Neuroticism: Extraversion Openness.

^c^PASS-process: Perceived Positive Appraisal Style Scale—process focused.

^d^COPE: Coping Orientation to Problems Experienced Inventory.

^e^CERQ^:^ Cognitive Emotion Regulation Questionnaire.

^f^PASS: Perceived Positive Appraisal Style Scale.

**Table 5:** List of self-report questionnaires used to assess secondary resilience and risk factors. Please note that only the original publications are cited here and not the validation studies of versions translated into the 4 study languages. Self-developed questionnaires are provided at the Center for Open Science [52].

Name	Construct
Anxiety Sensitivity Index	Beliefs of negative implications of anxiety experiences (18 items) [68].
Bem Sex Role Inventory	Subjective gender roles (30 items) [69,70].
Body Awareness Questionnaire	Subjective attentiveness to nonemotive body processes, such as the sensitivity to body cycles and rhythms, the ability to detect small changes in normal functioning, and the ability to anticipate bodily reactions (18 items) [71].
Connor-Davidson Resilience Scale	Subjective ability to cope with stress (10 items) [72].
Dimensional Anhedonia Rating Scale	Multiple facets of hedonic function such as desire, motivation, effort, and consummatory pleasure across hedonic domains (17 items) [73].
Emotion Regulation Questionnaire	Habitual use of the emotion regulation strategies “cognitive reappraisal” and “expressive suppression” (10 items) [74].
Green Space Questionnaire and geographic information	Self-developed questionnaire assessing the degree to which participants have access to and make use of green spaces (parks and forests) in their living environment. In combination, geographic analysis of a participant’s address data is used to determine the degree of green space in their living environment (12 items).
Maltreatment and Abuse Chronology of Exposure	Abuse and neglect during development (52 items) [75].
Perceived Social Status Scale	Subjective socioeconomic status by means of a drawing of a ladder with 10 rungs, described to represent where people stand in society. Participants are instructed to indicate the rung that best represents where they stand on the ladder. In addition, the same question is asked for the dimensions of academic and occupational status [76].
Ruminative Thought Style Questionnaire	Components of ruminative thinking including problem-focused thoughts, counterfactual thinking, repetitive thoughts, and anticipatory thoughts (15 items) [77].
Sensitivity to Punishment and Sensitivity to Reward Questionnaire	Tendency for aversive and appetitive behavior (48 items) [78].
State Trait Anxiety Inventory	Symptoms of anxiety as a state and as a general trait (40 items) [79].
Toronto Alexithymia Scale	Deficiency in understanding, processing, or describing emotions (20 items) [80].

#### Neuropsychological Measures

The neuropsychological test battery included paper-pencil paradigms such as the Trail Making Test [81,82] to assess visual attention and task switching speed, the Hamburg-Wechsler-Intelligenztest für Erwachsene (HAWIE) Matrices to assess nonverbal logical reasoning [83], and the HAWIE Digit Symbol Test to assess processing speed [83]. Furthermore, a computer-based paradigm, the Stab/Flex task [53,54], was administered to assess cognitive flexibility (refer to section 1 and Figure S1 in Multimedia Appendix 2).

### Neuroimaging

#### Magnetic Resonance Imaging Data Acquisition

At all sites except for Warsaw, brain imaging data were acquired using identical models of 3 Tesla MAGNETOM Prisma systems (Siemens Healthineers) with 32-channel head coils (at TAU 64-channel head coil) using the following settings: multiband gradient-echo echo planar imaging (EPI) sequences (repetition time [TR]=800 ms, time to echo [TE]=37 ms, flip angle=52°, field of view [FOV]=208 mm, voxel size=2.0 × 2.0 × 2.0 mm, 72 slices, multiband acceleration factor=8, and phase-encoding direction=posterior to anterior [PA]) from the Center for Magnetic Resonance Research, University of Minnesota, as adopted from the Human Connectome Project, were used for blood oxygen–level dependent fMRI [84]. Before each task, a pair of blip-up and blip-down EPI sequences were acquired (TR=8000 ms, TE=66 ms, flip angle=90°, FOV=208 mm, and voxel size=2.0 × 2.0 × 2.0 mm), 1 with an anterior to posterior phase-encoding direction and 1 with a PA phase-encoding direction. Furthermore, a T1-magnetization-prepared rapid gradient echo (MPRAGE) sequence (TR=2500 ms, TE=2.22 ms, flip angle=8°, FOV=256 mm, and voxel size=0.8 × 0.8 × 0.8 mm), a fluid-attenuated inversion recovery (FLAIR) sequence (TR=9000 ms, TE=83 ms, flip angle=150°, FOV=220 mm, and voxel size=0.7 × 0.7 × 3.0 mm), and 2 diffusion-weighted imaging sequences (1. TR=3600 ms, TE=92 ms, flip angle=78°, FOV=210 mm, voxel size=2.0 × 2.0 × 2.0 mm, 50 directions b=1000, 50 directions b=2000, 5 b0 volumes, multiband acceleration factor=3, phase-encoding direction=anterior to posterior and 2. TR=3600 ms, TE=92 ms, flip angle=78°, FOV=210 mm, voxel size=2.0 × 2.0 × 2.0 mm, 6 directions b=2000, multiband acceleration factor=3, phase-encoding direction=PA), adopted from the UK Biobank scan protocol [85], were acquired.

In Warsaw, a 3 Tesla MAGNETO Trio system was used. There, multiband gradient-echo EPI sequences were acquired with the following settings: TR=1410 ms, TE=30.4 ms, flip angle=56°, FOV=210 mm, voxel size=2.5 × 2.5 × 2.5 mm, 60 slices, multiband acceleration factor=3, and phase-encoding direction=PA. Blip-up and blip-down EPI sequences before each task (identical settings as other sites, except for voxel size=2.5 × 2.5 × 2.5 mm), T1-MPRAGE (TR=1100 ms, TE=3.32 ms, flip angle=7°, FOV=256 mm, voxel size=1.0 × 1.0 × 1.0 mm), a FLAIR sequence with identical settings as described in the previous paragraph, and 2 diffusion-weighted imaging sequences (identical settings as above, except TE=97 ms) were acquired as well.

Head movement was restricted by foam pads and a tape on the forehead. All task paradigms were presented using the software Presentation (Neurobehavioral Systems) [86] on a monitor placed behind the scanner bore via a mirror that was fixed on the head coil.

#### Reward Sensitivity

To capture neural responses during reward and loss anticipation, an adapted version of the monetary incentive delay task [87] was used, as described in detail by Kampa et al [37]. Participants were instructed that they can win or lose a small amount of money if they pressed a button fast enough as soon as the target stimulus appeared on the screen. Before the target appeared, participants were presented with a cue for 2 seconds, which indicated whether they could win or lose money in the current trial (+€3, ₪12, or zł12; +€0.5, ₪2, or zł2; ±0; −€0.5, ₪2, or zł2; and −€3, ₪12, or zł12). The conversion rates at the time of study enrollment were €1=US $1.17, ₪1=US $0.29, and zł1=US $0.26. The cue was followed by a jittered anticipation phase of 2 to 2.5 seconds, after which participants had to press a button as soon as a target stimulus (white star) appeared on the screen. Each trial ended with a 2-second numeric feedback on the participant’s trial outcome and the overall gain. To ensure that the reward experience did not differ between participants depending on task performance, an adaptive algorithm was applied that changed the target duration for the participant within each condition based on their past performance. If the participant’s hit rate was <66%, the target duration was increased by 25 ms; otherwise, it was reduced by 25 ms. Reaction times and hit rates were collected as behavioral outcomes. A graphical depiction of the task design is provided in Figure S2 in Multimedia Appendix 2.

#### Differential Fear Conditioning

We used the fear conditioning part of a safety learning and memory paradigm, as described by Kampa et al [37]. Three different geometrical shapes (squares, circles, and triangles) served as the CS. Electrotactile stimuli delivered to the back of the right hand were used as the unconditioned stimuli. Two background images of different conference rooms were inherited from the original paradigm in which they served as context variables. Each background image showed a screen on which the CS were depicted to make them part of the scene in a naturalistic way (Figure S3 in Multimedia Appendix 2). Before the main experiment, a short training session was given during which the different stimuli and the rating scale were shown, but no electric stimuli were delivered. During the experiment, 2 of the CS were paired with an unconditioned stimulus in 100% of the trials. These stimuli worked as CS+. The other stimulus (CS−) was never paired with an unconditioned stimulus. Each CS trial lasted for 6 seconds. During the first 4.5 seconds of the trial, participants rated their fear of receiving an electric stimulus between 1 and 100 using a visual analog scale at the bottom of the screen. The intertrial intervals lasted from 9 to 15 seconds. The background images and stimuli were counterbalanced across participants.

The stimulus intensity was calibrated beforehand using a calibration procedure developed to reach a stimulus level that was highly unpleasant but not painful. Participants received an initial stimulus at the lowest level (1) and were asked to rate its severity on a scale ranging from 1 (not unpleasant at all) to 5 (painful). The desired rating was 4. Stimulus intensity was adapted after each stimulus according to a predefined scheme (Figure S4 in Multimedia Appendix 2).

Stimulation was performed using 2 sticky electrodes (INVISATRACE Adult electrocardiography (ECG) Electrode) placed on the back of the hand. Except for the DCCN, all sites used a Digitimer DS7A stimulator. One electrical stimulation consisted of a train of 3 square-wave pulses of 2-ms duration each, with an interval of 50 ms apart from each other. The electric potential was 400 V, and the current varied with the calibration, starting at a minimum of 10 mA. At the DCCN, the electric stimuli were delivered using an Innostim Tens 2000 (formerly named MAXTENS2000, Bio-Protech Inc) [88]. The stimulus duration was 200 ms, and the intensity ranged between 0 V to 40 V and 0 mA to 80 mA.

At TAU, the electric stimulus was replaced by white noise with a duration of 50 ms. The volume was maximized to create a startle response. All the other conditions (background and reinforcement schemes) remained the same. A pilot study at TAU revealed similar activation patterns as the typical activation patterns found using an electric stimulus.

#### Situation-Focused Volitional Reappraisal

In this task, participants were instructed to positively reinterpret or just view photographs that are either negative, positive, or neutral and to rate their affective state on a nonverbal scale. The paradigm has been previously described by Kampa et al [37] and was adapted from the study by Kanske et al [89]. Stimuli were selected from the International Affective Picture System and EmoPics [90] based on normative ratings of valence and arousal [91]. In a fully balanced, 3-by-2 factorial design, the 3 types of picture valences were combined with either situation-focused reappraisal or viewing the pictures as a control condition (Figure S5 in Multimedia Appendix 2).

#### Implicit Emotion Processing

To assess neural responses during implicit emotion processing, we used an adaptation of the face matching task [48,49]. In each trial, participants were presented with a trio of pictures and were instructed to select the matching pair by pressing a button. In the emotion condition, the trios contained grayscale photographs of Ekman faces [92] with angry or fearful expressions, counterbalanced for sex and emotion valence. In the control condition, the trios contained geometric shapes (circles, horizontal ellipses, and vertical ellipses). Four blocks per condition were presented in alternation. Each block consisted of 1 instruction (2 seconds) and 6 trials (5 seconds each; refer to Figure S6 in Multimedia Appendix 2 for the task design).

#### Resting State

A resting-state scan was acquired before and after the stress task, during which participants were instructed to keep their eyes open and to focus on a fixation cross on the screen.

#### Social Stress

To examine brain activation and cortisol levels in response to stress, an adaptation of the ScanSTRESS paradigm was used [93-95]. In this task, the participants were instructed to perform mental rotation and arithmetic subtraction exercises. During the performance, task speed and difficulty were automatically adjusted so that the participants fail most of the tasks. Furthermore, participants were presented with a live video screen showing the face of the experimenter observing and giving negative nonverbal feedback on the performance. Thus, the task involves both social evaluative threat components (verbal and nonverbal feedback from the experimenter) and uncontrollable components (task difficulty, time constraints, and mock feedback of poor performance). The original version of the task is composed of 2 runs, both containing control (no feedback and no video) and stress (feedback and live video of experimenters) blocks, 1 run before and 1 after negative verbal feedback by the experimenter. Here, we used the adapted version by Sandner et al [47], in which all stress blocks are presented in one run and all control blocks in another run, both presented before and after negative verbal feedback by the experimenter. However, we shortened this version by discarding the control runs and using only stress runs. Specifically, a shorter practice run of stress blocks was presented first, followed by negative verbal feedback, after which a full run of stress blocks was presented during scanning. Brain responses to the stress paradigm are estimated by comparing task blocks with baseline (fixation cross). An overview of the task design is provided in Figure S7 in Multimedia Appendix 2.

### Biosamples

Blood was collected for the purpose of DNA extraction from whole blood (to determine participants’ genotype and DNA methylation patterns using next-generation sequencing) and—at 2 sites only—for blood plasma preparation (to perform proteome analyses using immunoassays). Additional blood collected at 1 site was used to determine interleukin (IL)-10, IL-6, tumor necrosis factor α (TNF-α), and C-reactive protein levels. Stool samples were collected for the purpose of gut microbiome analysis by 16S rRNA profiling. Saliva samples were collected to determine cortisol and α-amylase levels. Details of the analyses performed will be provided in individual publications.

### Ambulatory Assessments

During monthly burst weeks (6 days) in phase 1, EMA and EPA were measured.

### EMA Technique and Design

At the beginning of each burst week, participants received a smartphone (Motorola Moto E6 Play) with the app “RADAR-active RMT (Remote Assessment of Disease and Relapse Remote Monitoring Technology)” for EMA data collection [96]. Questionnaires were sent 10 times per day during the participants’ usual wakening hours using push notifications (“beeps”). Notifications were scheduled to appear within 90-minute blocks semirandomly, that is, for all participants, the notifications appeared at the same time. Similarly, 2 notifications never occurred closer than 15 minutes to each other (refer to Table S2 in Multimedia Appendix 2 for the beep schedule). The participants received a reminder notification 5 minutes after the initial notification. The EMA questionnaire remained available for 10 minutes after the initial push notification. Every beep questionnaire (approximately 3 minutes) included in-the-moment self-assessments of mood, (virtual) social context, physical context, event appraisal, substance use, and anticipation of pleasure. In addition, participants were instructed to start a separate morning questionnaire (approximately 1 minute) about the previous night’s sleep immediately after waking up. Immediately before going to bed, participants were instructed to start an evening questionnaire (approximately 2 minutes) about the evaluation of the day, the most negative and positive event of the day, stress anticipation for the upcoming day, and whether the questionnaire influenced their mood during that day. All EMA items are shown in Figures S8 and S9 in Multimedia Appendix 2.

### EPA Technique

EPA was measured for 23 hours a day during each burst week using the Chill+ wristband developed by Interuniversity Microelectronics Centre [97]. The wristband measures the photoplethysmography-based heart rate, galvanic skin response, skin temperature, and movement through a 3-axis accelerometer and 3-axis gyroscope. Furthermore, participants were instructed to press a button on the wristband to actively report stressful events.

### Procedure

#### Overview

Participants underwent a screening for inclusion criteria, 2 assessment days at baseline (Table 6), and longitudinal follow-up assessments (phase 1 and phase 2; Multimedia Appendix 4). Before each on-site appointment, a short screening interview about potential COVID-19 symptoms including measurements of or questions regarding body temperature was conducted to minimize the risk of transmission. The participants and experimenters wore filtering facepiece masks (level 2) or surgical masks, used disinfectants, and maintained a distance of at least 1.5 m.

**Table 6:** Procedure steps at baseline days 1 and 2. Note that, before each functional magnetic resonance imaging sequence, a field map scan was acquired. The total duration of the imaging battery was approximately 2 hours.

Procedure step and task/interview/test				Self-ratings		Duration^a^ (mm:ss)
**Day 1**						
	**On-site screening interviews**					
		Informed consent	—^b^		—	
		MINI^c^	—		—	
		Drug screening	—		—	
	**Neuropsychological tasks**					
		StabFlex	—		—	
		Trail making test	—		—	
		Digit symbol test	—		—	
		Matrices test	—		—	
	**Biological samples**					
		Blood	—		—	
		Stool instruction	—		—	
	**Postassessment interviews**					
		Longitudinal schedule	—		—	
		Online questionnaire briefing	—		—	
		Emotional disturbances interview	—		—	
**Day 2**						
	**Pre MRI^d^**					
		Drug screening	—		—	
		MRI training	—		—	
	**MRI battery**					
		Saliva sample 1	Perceived stress		—	
		Reward sensitivity task	—		08:26	
		Saliva sample 2	Perceived stress		—	
		Fear conditioning calibration	—		—	
		Fear conditioning task	—		12:10	
		Saliva sample 3	Perceived stress		—	
		T1 weighted image	—		06:54	
		Reappraisal task	Performance		13:06	
		Saliva sample 4	Perceived stress		—	
		Faces matching task	—		04:34	
		FLAIR^e^	—		02:44	
		Preresting state	Perceived stress		—	
		Resting state 1	—		07:10	
		Saliva sample 5	Perceived stress		—	
		ScanSTRESS training	—		—	
		ScanSTRESS task	—		06:26	
		Post ScanSTRESS	Perceived stress		—	
		Resting state 2	—		07:10	
		Saliva sample 6	Perceived stress		—	
		DTI^f^	—		06:32	
		Out of scanner	—		—	
	**Post MRI**					
		Saliva sample 7	Perceived stress		—	
		MRI exit interview	—		—	
		EMA^g^/EPA^h^ briefing	—		—	
		Saliva sample 8 (20 minutes after Saliva sample 7)	Perceived stress		—	
		Saliva sample 9 (20 minutes after Saliva sample 8)	Perceived stress		—	
		MRI debriefing	—		—	

^a^Durations are reported in the format minutes:seconds and are only mentioned for the MRI tasks, which had exactly the same durations for all participants.

^b^Self-ratings are only mentioned where applicable.

^c^MINI: Mini-International Neuropsychiatric Interview.

^d^MRI: magnetic resonance imaging.

^e^FLAIR: fluid-attenuated inversion recovery.

^f^DTI: diffusion tensor imaging.

^g^EMA: ecological momentary assessment.

^h^EPA: ecological physiology assessment.

#### Recruitment and Prescreening

Participants were recruited via web-based advertisements in mailing lists and on social media platforms. After receiving brief information about the study purpose, methods, and prerequisites, participants were invited to anonymously fill in a web-based screening survey via the platform SoSci Survey [98], in which the eligibility criteria (Table 1) of the study were checked using an automated algorithm. When starting the screening survey, participants were instructed to generate an anonymous code, which they later provided to the study staff in case of their inclusion, to link their screening data to their participant ID. The screening data of nonincluded participants remained anonymous. After completing the web-based screening questionnaire, eligible participants received an invitation to participate and a request to contact their study site. The complete study information material was then sent to the participants via email, and an on-site appointment was made.

#### On-site Screening

During the on-site appointment, the participants received verbal information about the study and provided written informed consent. Then, further inclusion criteria were assessed in a standardized interview with trained researchers (Mini-International Neuropsychiatric Interview [MINI] [51]). Afterward, a urine-based drug screening test (SureStep Multi-Drug One Step Screen Test Panel, Innovacon Inc) for amphetamine, barbiturates, benzodiazepines, buprenorphine, clonazepam, cocaine, fentanyl, heroin, ketamine, cannabis, methadone, methamphetamine, methylenedioxymethamphetamine, morphine, opiate oxycodone, phencyclidine, propoxyphene, tramadol, and tricyclic antidepressants was administered, after which participants who were included started with the baseline assessment (baseline day 1).

#### Baseline Day 1

Baseline assessments were distributed across 2 appointments on 2 days (Table 1).

##### Neuropsychological Assessments

During neuropsychological assessments, 1 participant per session was assessed by 1 study assistant. The participant was placed at a desk with a computer in a room with minimized potentially distracting stimulation (eg, noise, visual distractions, and other people). Telephones were muted or in airplane mode. The first task (Stab/Flex) was presented on the computer, and all other tasks were instructed verbally and executed in a paper-and-pencil format (refer to the *Materials* section).

##### Blood Sampling

One blood sampling was performed at baseline day 1 or baseline day 2, and another one was performed at month 6 (Multimedia Appendix 4). Nine milliliters of blood (10 at DCCN) was drawn from each participant into an ethylenediaminetetraacetic acid (EDTA) tube (red monovette; Sarstedt) and stored as whole blood at −20 °C or colder until assay of DNA and DNA methylation. At the study sites NIC and DCCN, an additional 9 mL (NIC) or 10 mL (DCCN) of blood was sampled into EDTA tubes for proteomic analyses. At these sites, all blood was drawn between 12:30 PM and 3:30 PM, and participants arrived at least 5 hours sober to limit the influence of metabolism or diurnal oscillations on proteomics measurements. Blood samples for the proteomics assay were centrifuged, and serum was divided into 8 to 16 aliquots (depending on volume), which were stored at −80 °C until assay. At TAU, 2 additional tubes (1 EDTA and 1 VACUETTE TUBE 3.5 mL coagulation activator tube Serum Separator Clot Activator) of blood samples were taken between month 1 and month 6 to derive IL-10, IL-6, TNF-α, and C-reactive protein.

##### Stool Sampling

Stool samples were collected using an OMNIgene-gut feces kit (OM-200, DNAgenotek). Participants received a test kit, an instruction sheet about the collection procedure, the Bristol Stool Scale [99], and verbal instructions from the test leader. Participants were instructed to collect the stool sample as close as possible to the appointment, to take several small samples from different locations in the stool material, to fill out the Bristol Stool Scale, and to store the sample in a dark place without direct sunlight. Participants brought the sample to the study center at the next appointment, where it was stored at −20 °C until the assay of the gut microbiome, or, at DCCN, directly shipped to the laboratory processing the microbiome.

##### Postassessment Procedures

After the baseline day 1 assessments, a personalized schedule was created with the participant, indicating the upcoming magnetic resonance imaging (MRI) appointment and timings of the upcoming phase 1 and phase 2 assessments. Furthermore, participants received all necessary information about the web-based questionnaires and were introduced to the web-based survey platform SoSci Survey [98] by filling out a dummy questionnaire. Finally, to ensure the participant’s well-being, they were asked if they have experienced emotional disturbances triggered by any questions asked during the preceding session in a standardized interview. If they reported emotional disturbance and a need for help, they were directed to a clinician associated with the study.

#### Baseline Day 2

##### Overview

At baseline day 2, neuroimaging and a briefing about ambulatory assessments were performed. Before testing, participants returned their stool sample (except for DCCN), and another urine-based drug screening test (refer to the description for baseline day 1) was conducted. Participants with a negative test result then received a brief training session on MRI paradigms. During the training, they were given an on-screen presentation of the tasks, and the experimenter explained the tasks and asked questions to ensure that the participants understood the instructions. To account for diurnal or metabolism-related variations in cortisol levels, all scanning took place between 12:30 PM and 5 PM. Participants were instructed to get up from bed at least 4 hours before the appointment; not to eat, smoke, or drink beverages containing caffeine or sugar at least 2 hours before starting the MRI; to refrain from physical exercise that day; and not to drink alcohol within 24 hours before the appointment. They were reminded of these instructions via email before the appointment.

##### Physiological and Subjective Measures During Neuroimaging

During all fMRI sequences, the participants’ heart rates were assessed using a wireless pulse oximeter (Siemens Healthineers). Furthermore, to assess salivary cortisol levels in response to the MRI tasks, 9 saliva samples were collected before, in between, and after the scanning sequences using Salivette collection kits (Sarstedt). For each saliva sample, the participant’s subjective level of distress was assessed on a scale from 0 (not stressed) to 10 (extremely stressed). Before scanning, the participants were introduced to the saliva sampling technique while donating their first saliva sample. The participant received a plastic tube containing a cotton swab and was instructed to put the cotton swab into their mouth without touching it with their fingers. They were instructed to moisten the cotton swab for 1 minute and to put it back into the plastic tube. This procedure was repeated for 8 times, of which the participant was inside the scanner 5 times (Table 6).

##### Neuroimaging

When placed in the MRI scanner, the participants were provided with earplugs. To respond to tasks during functional MRI scans, participants operated a 4-button Inline Fiber Optic Response Pad (Current Designs; home-designed system in Warsaw [100]) with their right hand. Further, an electrode for the fear conditioning task was attached to the back of the right hand. A wireless pulse oximeter was attached to the index finger of their left hand. Via a mirror placed on the head coil, they were presented with the visual stimulation of the tasks on a monitor placed behind the scanner bore. Before and after each task, the test leader provided verbal instructions via an intercom system and received feedback from the participant. Specific instructions were repeated verbally on the screen and by the test leader before each task. An overview of the procedure steps during neuroimaging and further details of the tasks are provided in Table 6 and Multimedia Appendix 2. After scanning, participants were asked to fill in an MRI exit interview questionnaire (paper-pencil), which asked about the experiences and potential difficulties with the fMRI tasks.

##### EMA and EPA Briefing

After scanning, the participants were thoroughly briefed on the EMA and EPA devices and procedures. Contraindications for using the wristband collecting EPA (skin disease around the wrist area, wounds or skin allergies, and medication with phototoxic side effects) were ruled out. The devices (mobile phone, wristband, and chargers) were handed to the participants, and their functions were explained. Furthermore, the purpose of EMA and EPA, the app, and questionnaires were explained, and it was discussed with the participant how to ensure that beeps are not missed in everyday life. Further details are provided in the *Materials* section and Multimedia Appendix 2.

##### Post-MRI Debriefing

After donating their last saliva sample, participants were debriefed about the stress task (refer to the *Materials* section) and were informed that the task was programmed to adapt to the participant’s performance and induce stress, instead of measuring their cognitive performance. Furthermore, identical to baseline day 1, participants were interviewed about emotional disturbances triggered by any questions asked during the preceding session in a standardized fashion. If they reported emotional disturbance and a need for help, they were offered counseling by a clinician associated with the study.

#### Longitudinal Phase 1

In the first week after the baseline day, a baseline battery of questionnaires was administered on the web. Longitudinal phase 1 spanned from month 1 to month 6 and contained the web-based monitoring for the FRESHMO paradigm [13]. In biweekly web-based questionnaires, stressor exposure, mental health problems, and process-based PAS (PASS-process) were assessed on the web. For this, participants received a link to the SoSci Survey platform at the beginning of each web-based monitoring week and had 1 week to complete the questionnaire. Furthermore, 1-week EMA and EPA assessments took place in the second week of each of the 6 study months, for which participants received 10 beeps per day (Multimedia Appendix 4).

Before and after the EMA and EPA weeks, the participants came to the laboratory to pick up or return the devices used for EMA and EPA (smartphones and wristbands). During these appointments, the participants were again interviewed about potential emotional disturbances to ensure their well-being. At months 3 and 6, further stool samples were collected. At month 6, a second blood sample was collected (Multimedia Appendix 4).

#### Longitudinal Phase 2

In longitudinal phase 2, which spanned from month 7 to 9, assessments of stressor exposure, mental health problems, and PASS-process took place only once a month, and no EMA and EPA was conducted. The final questionnaire battery at month 9 contained additional assessments of the resilience factors (Multimedia Appendix 4).

#### Remuneration

Complete participation in all assessments was remunerated with €290 (₪1224 in Tel Aviv and zł1200 in Warsaw). Participants could win about €10, ₪40, or zł40 on average during the reward task in the neuroimaging battery. Furthermore, those who completed all assessments until month 6, week 3, were included in a lottery to win 1 out of 3 additional awards of €100, ₪400, or zł400. To maintain compliance throughout the longitudinal assessments, money was disbursed in tranches at different timepoints throughout the study (Table S3 in Multimedia Appendix 2). The conversion rates to at the time of study enrollment were €1=US $1.17, ₪1=US $0.29, and zł1=US $0.26.

## Results

Participant inclusion started in October 2020, and data acquisition was completed in June 2022. A total of 249 participants completed baseline days 1 and 2, whereas 209 completed the EMA part of longitudinal phase 1, and 153 completed longitudinal phase 2. At the time of manuscript submission, preprocessing and quality checks of the data are ongoing. First results are expected to be published by the end of 2023.

## Discussion

### Aims of This Study

DynaM-OBS targets a gap in resilience research: providing a dense longitudinal database to assess resilience as a long-term outcome and the underlying resilience processes. This approach and framework offer new possibilities to test current hypotheses about putative resilience factors as well as to explore novel ones. Specifically, our main hypothesis is that a PAS is both contemporaneously and prospectively predictive of resilience, operationalized as SR. Moreover, we expect several secondary predictors of resilience, ranging from specific brain network configurations during acute stress to high reward sensitivity, threat safety discrimination, and volitional reappraisal processes, among others.

### Strengths and Limitations

Despite its strengths, which are mainly reflected in the study’s richness of longitudinal and multimodal data on resilience factors, processes, and outcomes, DynaM-OBS also has several limitations. Although we were trying to be exhaustive in our assessment of stressors (including potential life events, a large number of microstressors in a wide range of life domains, and pandemic-related stressors), we cannot exclude the possibility that some relevant types of adversity were missed. For example, we did not collect information on parental traumatic exposure, which may transmit to children, and did not ask specific questions on the exposure of participants to stressors that only became more apparent over the course of data collection (eg, Russia-Ukraine war). Moreover, because of the multicenter nature of our study, we could not entirely rely on previously validated questionnaires for each study site’s language and therefore translated some questionnaires ourselves, without validating them. This may pose a threat to the construct equivalence and validity between the nonvalidated and validated versions of the questionnaires. Another consequence of the multicenter nature of DynaM-OBS is that different COVID-19 restrictions applied at the different sites. Among other things, this led to the interruption of participant inclusion at Charité–Universitätsmedizin Berlin from mid-December 2020 to the end of February 2021, although all other study sites continued participant inclusion. For the interested reader, we present an overview of the general COVID-19 situation at all sites over the course of data collection in Multimedia Appendix 1.

### Conclusions

Despite these limitations, the dense investigation of the most important resilience factors and resilience processes and the multi-modal database generated in DynaM-OBS have the potential to serve as a foundation for future studies on interventions and trainings aiming to enhance resilience factors and adaptive processes in at-risk individuals.

## Appendix

Multimedia Appendix 1 Overview of the COVID-19–related situation during data acquisition per country.

Multimedia Appendix 2 Supplementary information.

Multimedia Appendix 3 Overview of questionnaire validations for the different study languages.

Multimedia Appendix 4 Overview of the measures used and the days (d), weeks (w), and months (M) from baseline at which they are assessed (x).

## Abbreviations

CS: conditioned stimuli
DCCN: Donders Centre for Cognitive Neuroimaging
DynaCORE-L: The DynaMORE Longitudinal Study on Psychological Resilience to the Mental Health Consequences of the Corona Crisis
DynaM-INT: DynaMORE intervention study
DynaM-OBS: Dynamic Modelling of Resilience–Observational Study
DynaMORE: Dynamic Modelling of Resilience
EAN: ethics approval number
EDTA: ethylenediaminetetraacetic acid
EMA: ecological momentary assessment
EPA: ecological physiological assessment
EPI: echo planar imaging
FLAIR: fluid-attenuated inversion recovery
fMRI: functional magnetic resonance imaging
FOV: field of view
FRESHMO: frequent stressor and mental health monitoring
GHQ: General Health Questionnaire
HAWIE: Hamburg-Wechsler-Intelligenztest für Erwachsene
IL: interleukin
LORA: Longitudinal Resilience Assessment
MARP: Mainz Resilience Project
MINI: Mini-International Neuropsychiatric Interview
MPRAGE: T1-magnetization-prepared rapid gradient echo
NIC: Neuroimaging Center
OSF: Open Science Framework
PA: posterior to anterior
PAS: positive appraisal style
PASS-content: Perceived Positive Appraisal Style Scale—content focused
PASS-process: Perceived Positive Appraisal Style Scale—process focused
PASTOR: positive appraisal style theory of resilience
RF: resilience and risk factors
SR: stressor reactivity
TAU: Tel Aviv University
TE: time to echo
TNF-α: tumor necrosis factor α
TR: repetition time

## Data Availability

Self-generated questionnaires are available at the Center for Open Science [52].
