# Supplementary material for: Dynamic Modelling of Mental Resilience in Young Adults: Protocol for a Longitudinal Observational Study (DynaM-OBS)
Source: JMIR Res Protoc. 2023 Jul 4;12:e39817. doi: 10.2196/39817 (PMC10354714; doi:10.2196/39817)
Supplement: Multimedia Appendix 2 [file resprot_v12i1e39817_app2.docx]

Dynamic Modelling Of Mental Resilience In Young Adults - Protocol For A Longitudinal Observational Study

Supplementary Information

**Table of Contents**

[1 Stab/Flex Task 2](#_Toc137113484)

[2 Design of Neuroimaging Tasks 4](#_Toc137113485)

[2.1 Reward sensitivity 4](#_Toc137113486)

[2.2 Differential fear conditioning 4](#_Toc137113487)

[2.3 Situation-focused volitional reappraisal 6](#_Toc137113488)

[2.4 Implicit emotion processing (Faces task) 6](#_Toc137113489)

[2.5 Social Stress 7](#_Toc137113490)

[3 Sociodemographic Covariates 8](#_Toc137113491)

[4 Ambulatory Assessments 13](#_Toc137113492)

[4.1 EMA beep schedule 13](#_Toc137113493)

[4.2 EMA questionnaire 14](#_Toc137113494)

[5 Remuneration schedule 16](#_Toc137113495)

# Stab/Flex Task

The Stab/Flex task [53] was administered using the software Presentation® (Neurobehavioral systems, [https://www.neurobs.com](https://www.neurobs.com/)).

In this task, participants were presented with a cue in the middle of the screen and a digit between 1 and 9 (excluding 5) above and/or below the cue. The cue consisted of a circle on a vertical line. When the circle is in the upper half of the line, participants are instructed to respond to the number above the cue, when it is in the lower half, participants are instructed to respond to the number below the cue. Two tasks were assigned in a counter-balanced fashion: 1) to evaluate if the number is odd or even, 2) to assess whether the number is lower or higher than

5. Participants were told that the cue is always pointing towards one of the two numbers. One of these tasks (depending on randomization) was used as baseline task.

Before the two experimental blocks, 4-6 training blocks were given, during which participants first practiced the two tasks individually, and then both tasks together. During training sessions 1 to 3, feedback was given, while the following training sessions resembled the actual task and had no feedback. Participants were informed that the cue always pointed toward one of the two digits but that in some cases it might be difficult to determine. Further, instructions were to always respond as quickly as possible to the trials and to follow their first intuition in cases that seemed unclear.

The main experiment consisted of two blocks of 150 trials each. In 80% of the trials, only one number is displayed above the cue. These trials are used as baseline and the baseline task rule is applied. In the remaining 20% of trials, two numbers are displayed, and three different scenarios are programmed: 1) In one-third of the trials, the cue indicates to attend to the number above the cue. In this condition, participants must follow the baseline task while the number below the cue has to be ignored (distractor inhibition condition). 2) In another third of the trials, the cue points towards the number below. Participants then must switch to the other task and ignore the number above the cue (task switching condition). 3) In the last third of trials with two numbers on display, the cue is in the middle of the line, thus not pointing to either of the numbers (ambiguous condition). This condition is used to assess the rate of spontaneous switching (see Armbruster et al., 2012 for details). Trials have a fixed duration of 2000 ms. The stimuli are presented for 900 ms. Participants are able to respond during the whole period of the trial. No feedback about performance is given during the main task. The two blocks of the main experiment have a total duration of 10 minutes.


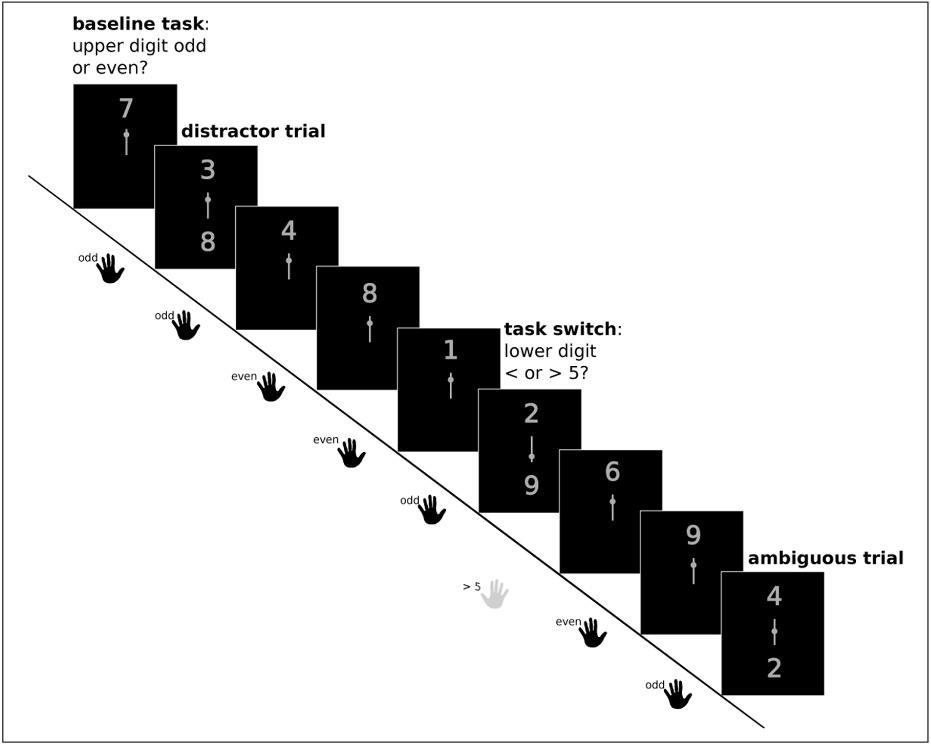


**Figure S1. Cognitive flexibility paradigm, adapted from Kraft et al [54].** Participants performed a baseline task (i.e., judging whether the presented digit is higher or lower than five) and responded with their left hand. In task switch trials, participants performed a different task (i.e., indicating whether the digit is odd or even) and need to respond with the right hand (highlighted in grey). The position of the small dot on the white bar in the center indicated whether participants were supposed to switch tasks. In ambiguous trials, the cue of whether to switch or not was ambiguous. These trials were used to estimate the rate of spontaneous switching. After a task switch, distractor, or ambiguous trial, participants continued to perform the ongoing task. Figure by Kraft et al. [54], adopted with permission from the authors.

# Design of Neuroimaging Tasks

## Reward sensitivity


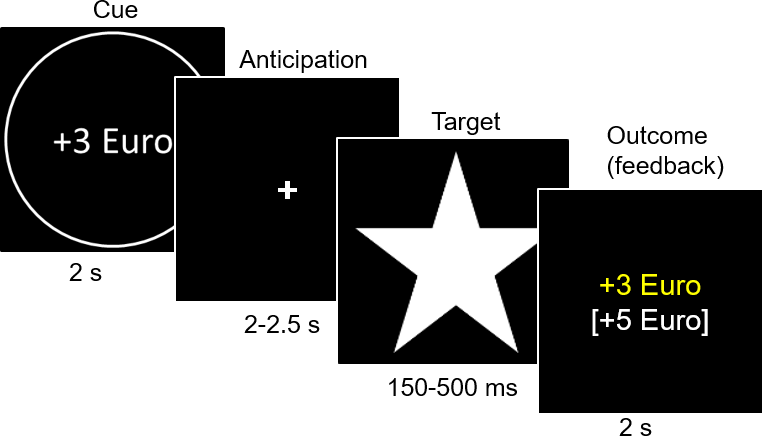


**Figure S2. Design of the reward sensitivity task.** Each trial started with a 2-seconds cue indicating the incentive condition (+3 €, +0.5 €, ±0 €, -0.5 €, -3 €), followed by an anticipation phase of 2 to 2.5 s. Subjects had to press the right button when the target (a white star) appeared on the screen. Target duration was adapted based on past task performance in a range from 150 and to 500 ms. Each trial ended with a 2- seconds numeric feedback on subjects’ trial outcome (gained amount in yellow or lost amount in red) and overall outcome (accumulated total amount). As an example, a correct +3 € trial is shown. Figure by Kampa et al. [37], adopted with permission from the authors.

## Differential fear conditioning


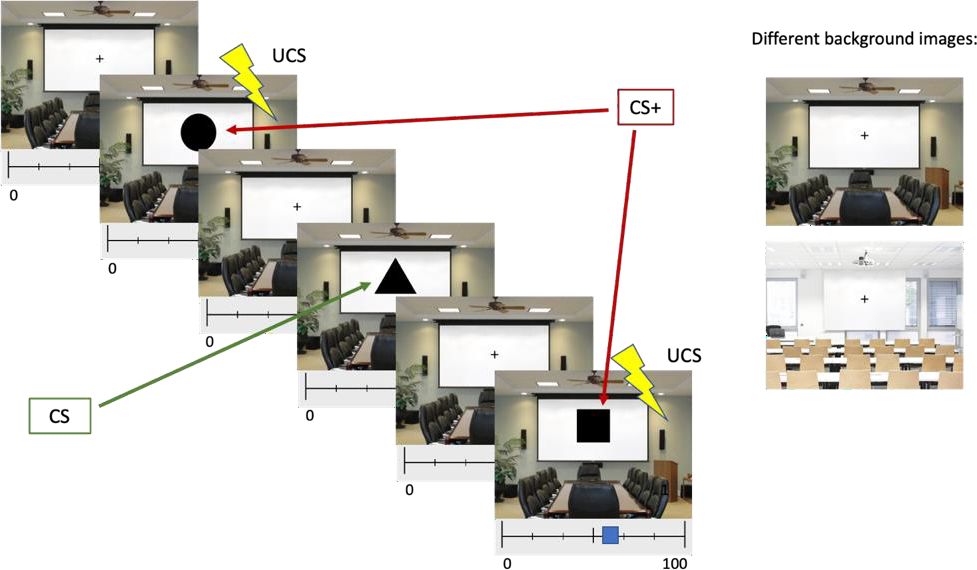


**Figure S3. Design of the differential fear conditioning task.** During the experiment, two of the CSs were paired with an electrical shock (UCS) in 100% of the trials, which was delivered through two electrodes on the back of the right hand. The other stimulus was never paired with a UCS. Each CS trial lasted 6 s. During the first 4.5 s of a trial, participants rated their fear of receiving a pain stimulus between 1 and 100 using a visual analog scale at the bottom of the screen. Inter-trial-intervals lasted 9 to 15 s. Background images and stimuli were counterbalanced across participants. Figure adapted from Kampa et al. [37] with permission from the authors.

| **stimulus level:** |  | **subjects rating (tick one)**  **(change for next level)** | | |  |
| --- | --- | --- | --- | --- | --- |
| **1 (start level)** | 1  (+2) | 2  (+1) | 3  (=) | 4  (=) | 5  (-1) |
|  | 1  (+2) | 2  (+1) | 3  (+1) | 4  (=) | 5  (-1) |
|  | 1  (+3) | 2  (+2) | 3  (+1) | 4  (=) | 5  (-1) |
|  | 1  (+3) | 2  (+2) | 3  (+1) | 4  (=) | 5  (-1) |
|  | 1  (+3) | 2  (+2) | 3  (+1) | 4  (=) | 5  (-1) |
|  | 1  (+3) | 2  (+2) | 3  (+1) | 4  (=) | 5  (-1) |


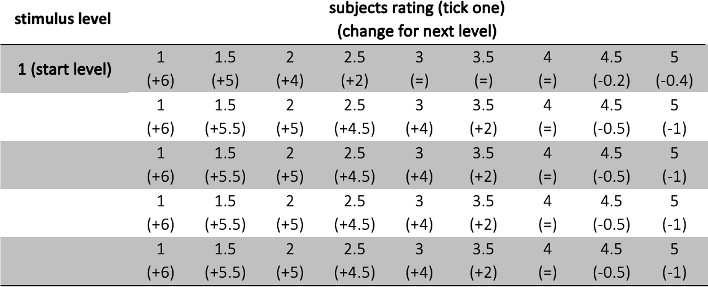


**Figure S4. Calibration scheme of the fear conditioning task.** Before the experiment, participants were presented with 5 trial shocks to reach an intensity that was rated as 4 = very unpleasant, but not painful. The first trial started at the lowest level (1) and participants were asked to rate their experience of the shock on a 5-point scale from 1 = “not unpleasant at all” to 5 = “painful”. Depending on their response, the intensity of the shock was adapted, for example, if participants rated the first shock with a “2”, the shock level was increased by two units (see scheme). For each step, the experimenter noted the new shock level in the left column, and another trial was executed. In total, 5 calibration trials were executed. The upper table shows the scheme for the DCCN, and the lower table shows the scheme for all other sites.

## Situation-focused volitional reappraisal


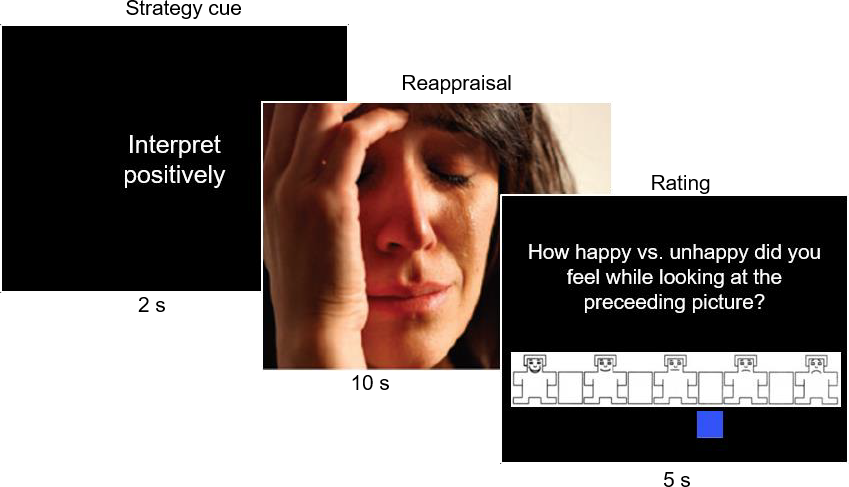


**Figure S5. Design of the Situation-focused volitional reappraisal task.** Subjects had to either make their emotional state more positive by positively reinterpreting a depicted situation (Reappraisal or R trials) or not (No Reappraisal or NR trials). Each trial started with a 2-second strategy cue word (“interpret positively” in R or ”view image” in NR trials), followed by a 10-second presentation of a positive (Pos), neutral (Neu), or negative (Neg) picture, during which the strategy was to be applied. Each trial ended with an emotional state rating from 0 to 5 for 5 s. As an example, a Neg/R trial is shown. The depicted image is retrieved from the EmoPicS (Wessa et al., 2010) and was already published in Kanske et al. (2011). It is used as a placeholder for emotional images here but is not part of the stimulus set of Task 5. Figure by Kampa et al. [37], adopted with permission from the authors.

## Implicit emotion processing (Faces task)


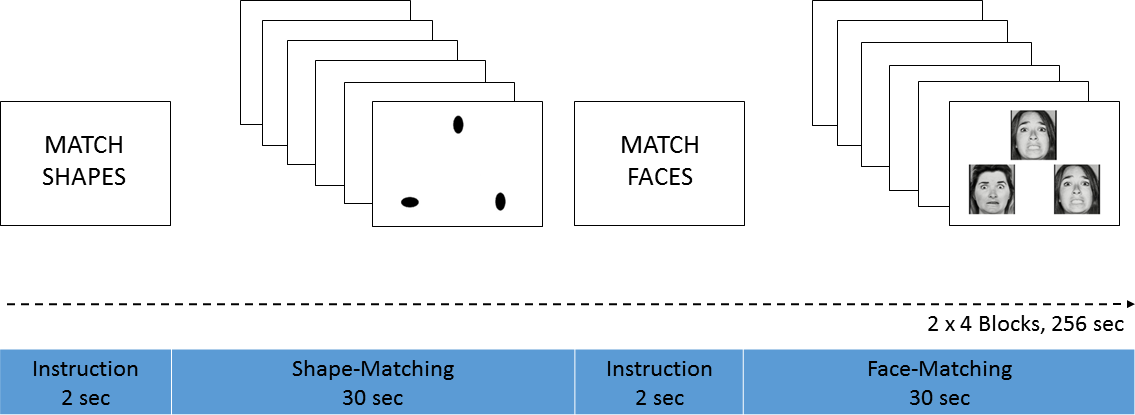


**Figure S6. Design of the implicit emotion processing task (Faces task).** Each block started with a brief instruction. In all conditions, participants were instructed to identify which one of the two stimuli at the bottom is identical to the stimulus at the top. During the shape-matching blocks, stimuli contained of vertical and horizontal ellipses, while during the face-matching blocks, stimuli consisted of photographs of male and female faces expressing anger or fear. Figure by Sacu et al. [55], adopted with permission from the authors.

## Social Stress


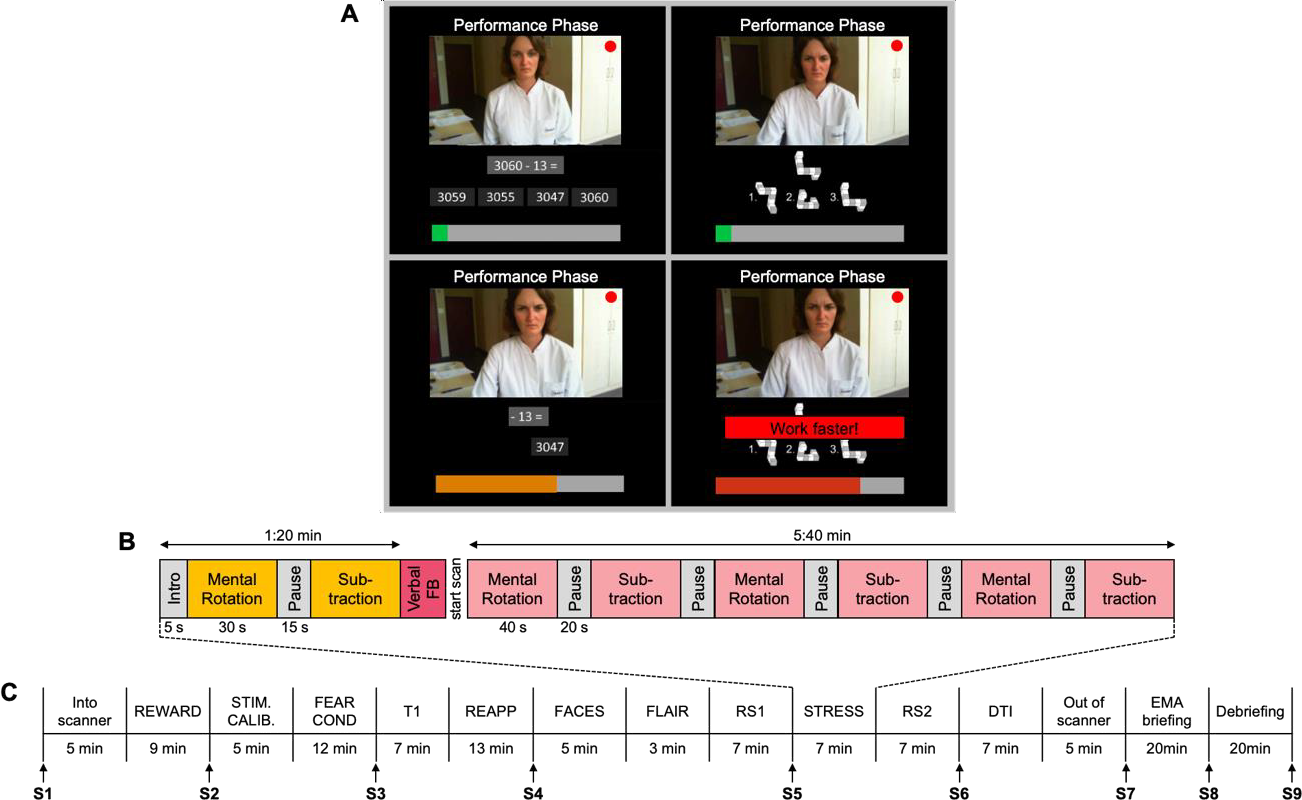


**Figure S7. Design of the Social stress task.** A: Example screenshots from the participant’s perspective during the performance phase of the mental rotation (left two screens) and the subtraction (right two screens) part of the stress task. B: Block design of the training phase (orange boxes) and performance phase (red boxes) of the stress task. The training phase took place without scanning and had identical visual instructions and feedback as the performance phase, which took place during scanning, after the experimenters gave verbal feedback to the participant. This feedback included telling the participant to show better performance during the scan, and that their performance was crucial for the usability of the data. C: Complete scanning battery and time points of saliva samples (S1-9), please note that the times of the procedure steps are approximates and can differ from this scheme.

# Sociodemographic Covariates

|  | **Construct** | **Answer options (if applicable)** |
| --- | --- | --- |
| **General sociodemographics, origin, & family history** | | |
|  | Age |  |
|  | Sex | Male; female; diverse |
|  | Gender | Male; female; diverse |
|  | Father: year of birth |  |
|  | Mother: year of birth |  |
|  | Father: medical conditions |  |
|  | Mother: medical conditions |  |
|  | Father: alive? If no: reason of death |  |
|  | Mother: alive? If no: reason of death |  |
|  | Age, gender, and medical conditions of potential siblings |  |
|  | Age, gender, and medical conditions of potential children |  |
|  | Mother tongue(s) |  |
|  | Other spoken languages |  |
|  | Nationality |  |
|  | Country of birth |  |
|  | Ethnicity of parents and grandparents | White skin/of Caucasian descent; of sub-Saharan African descent; of northern African/middle-eastern descent; of Asian descent; other; person unknown |
|  | Continent of birth of parents and grandparents | Africa; Antarctica; Asia; Europe, North America, Oceania; South America; person unknown |
|  | Being adopted |  |
|  | Being a twin |  |
|  | Are psychiatric disorders present in your family, and if yes, who is affected? | Mother, father, brother, sister, grandmother (mother), grandfather (mother), grandmother (father), grandfather (father) |
|  | Current living status | Alone; Together with my partner or family member(s); In a community (e.g. dorm rooms, nursing home, institution,...); At home (with parents and brothers/sisters) |
|  | Housing situation during chilodhood | Living with both parents; living with one parent; alternately living with mother/father; living with other relatives; looked after by welfare organizations; other (please specify) |
|  | Current living area | Rural; urban |
|  | Living area during childhood | Rural; urban |
|  | Inhabitants of village/town/city during growing up | < 10.000; 10.000-100.000; > 100.000 |
|  | Highest degree |  |
|  | Occupation |  |
|  | Working hours per week |  |
|  | Distance between home and work/university |  |
|  | Time needed to travel from home to work/university |  |
|  | Means of transportation usually used | Foot; bike; car; scooter; bus; tram; underground; (sub-)urban train; train; other (please specify) |
|  | Civil status | Single; unmarried but in a relationship, living together; unmarried but in a relationship, not living together; married/in a civil union, living together; married/in a civil union, not living together; divorced; widow; other (please specify) |
|  | Number of people in household |  |
|  | Number of people contributing to household income |  |
|  | Average household income |  |
|  | Personal income |  |
|  | Discipline of studies |  |
|  | Mother’s highest degree |  |
|  | Mother’s occupation |  |
|  | Father’s highest degree |  |
|  | Father’s occupation |  |
|  | Parents’ gross income per month |  |
|  |  |  |
|  |  |  |
|  |  |  |
|  |  |  |
|  |  |  |
|  |  |  |
|  | handedness |  |
| **Health and habits** | | |
|  | Physical exercise in past 3 months | Aerobic activity (running, walking, bicycle; swimming, dancing, or similar); anaerobic activity (yoga, pilates, stretching, or similar); strength exercises (lifting weights, resistance band, body weight); functional workouts (combination of strength and anaerobic); other (please specify); I did not perform any physical activity during the past 3 months; I did not perform any physical activity during the past 3 months due to physical issues |
|  | Frequency of physical exercises | < 1/month; 1-2/month; 1/week; 2-3/week; 4-5/week; 6-7/week; other |
|  | Perceived influence of physical exercise on physical health | Does not influence at all; Influences to a small extent; Influences to some extent; Influences to a high extent; Influences very much |
|  | Influence on physical health positive or negative | Positive; negative |
|  | Perceived influence of physical exercise on mental health | Does not influence at all; Influences to a small extent; Influences to some extent; Influences to a high extent; Influences very much |
|  | Influence on mental health positive or negative | Positive; negative |
|  | Amount of physical activity during work | I have a sedentary job/studies; I have a sedentary job/studies but walk/cycle to my job/university; I have a job for which I often have to move actively (walking the stairs, cycling, walking); I do heavy physical work; I am unemployed (looking for a job) |
|  | Mental activities in past 3 months | Yoga; Meditation/Mindfulness; Guided imagery; Biofeedback; Neurofeedback; Cognitive training; other (please specify); Don’t perform at all |
|  | Perceived influence of mental activity on mental health | Does not influence at all; Influences to a small extent; Influences to some extent; Influences to a high extent; Influences very much |
|  | Influence on mental health positive or negative | Positive; negative |
|  | Having ever taken performance-enhancing substances (if yes which) |  |
|  | Frequency of alcohol consummation | Never; very rarely; once in 2-3 months; once a month; once every two weeks; once a week; 2-3 times a week; 4-6 times a week; daily |
|  | Quantity of alcohol consumed every time | One drink; 2-3 drinks; 4-5 drinks; more than 5 drinks |
|  | Number of alcoholic drinks consumed during a weekday | 0 glasses; 1-2 glasses; 3-4 glasses; 5-6 glasses; > 6 glasses; I don’t know |
|  | Number of alcoholic drinks consumed during a weekend day | 0 glasses; 1-2 glasses; 3-4 glasses; 5-6 glasses; > 6 glasses; I don’t know |
|  | Having ever smoked | I smoke at the moment and started at the age of … ; I used to smoke and stopped at the age of … ; I have never smoked; Somebody in my house smokes |
|  | Number of cigarettes/cigarillos/cigars/… consumed on an average day |  |
|  | Frequency of cannabis consummation in the past year | Never; very rarely; once in 2-3 months; once a month; once every two weeks; once a week; 2-3 times a week; 4-6 times a week; daily |
|  | Consummation of other drugs in the past year | Cocaine/Ritalin without prescription; MDMA/Ecstasy; Ketamine; Mushrooms; LSD; Heroin/Crystal Meth; Prefer not to answer; None |
|  | Frequency of internet use | Never; 1 day/week; 2 days/week; 3 days/week; 4 days/week; 5 days/week; 6 days/week; 7 days/week |
|  | Average internet use on a workday | 1-2h; 2-3h; 3-4h; 4-6h; 6-8h; >8h |
|  | Average internet use on a weekend day | 1-2h; 2-3h; 3-4h; 4-6h; 6-8h; >8h |
|  | Type of birth | Vaginally; c-section; I don’t know |
|  | Having been breastfed as a baby | Yes, no, I don’t know |
|  | Duration of having been breastfed | < 3 months; 3-6 months; > 6 months; I don’t know |
|  | Having asthma | Yes, but not anymore (please specify from which age to which age you had asthma); Yes, and I still have it now (please specify how old you were when it started); I did not have it as a child, but I have it now; No; I don’t know |
|  | Having hayfever | Yes, but not anymore (please specify from which age to which age you had asthma); Yes, and I still have it now (please specify how old you were when it started); I did not have it as a child, but I have it now; No; I don’t know |
|  | Having eczema | Yes, but not anymore (please specify from which age to which age you had asthma); Yes, and I still have it now (please specify how old you were when it started); I did not have it as a child, but I have it now; No; I don’t know |
|  | Use of antibiotics | Yes, three or more times in the last three months; Yes, less than three times in the last three months; Yes, frequently (twice or more per year), but not in the last three months; Yes, seldom (less than twice per year), but not in the last three months; Never |
|  | Use of probiotics | Daily; Once per week; Once per month; Seldom; Never |
|  | Food allergies | None; Nuts; Seeds; Milk/dairy; Meat; Soy; Fish/shellfish; Eggs; Fibers (gluten); Fruit/vegetables; other (please specify) |
|  | Contact with farm animals during childhood | Daily; once a month; seldom; never |
|  | Current pets |  |
|  | Periods of staying outside of Europe/Israel in the past 10 years– if yes, where |  |
|  | Recent tonsillectomy/adenoidectomy | Tonsilectomy; Adenoidectomy; None; I don’t know |
|  | Having ever had a tick bite |  |
|  | Number of tick bites | 1; 2-5; 6-11; 12 or more |
|  | Having ever had a red circle on the skin after a tick bite | Yes, seen by me/my partner; Yes, seen by a general practitioner; Yes, seen by a specialist; No |
|  | Having ever been treated for Lyme disease | No; Yes – used antibiotics; Yes – didn’t use antibiotics |
|  | If having ever been treated for Lyme disease: When? Which symptoms? Treated by whom? |  |
|  | If having ever been treated for Lyme disease: presence of related health complaints |  |
|  | Presence of the following health complaints | Varicose veins; red or blue discolored legs or feet; difference in color between both legs or feet; difference in color between both ears; painful or thick joints; symptoms of a hernia; numbness of hands or feet; Arrhythmias; Memory problems; regular headaches; regular muscle aches; concentration problems |
|  | Having had a fungal infection (if yes, which kind) |  |
|  | General well-being | Excellent; very good; good; fine; bad |
|  | Health compared to people of same age | Less healthy/more often sick; equally healthy/equally often sick; more healthy/less often sick; a lot more healthy/a lot less often sick; I am never sick |
|  | Complaints about physical well-being during past three weeks | Always; usually; sometimes; occasionally; never |
|  | Complaints about mental well-being during past three weeks | Always; usually; sometimes; occasionally; never |
|  | Suffering or having suffered from any of the following medically diagnosed diseases (if yes, from when to when) | Diabetes mellitus; hypertension; elevated blood fat; heart attack; liver disease; kidney insufficiency; cancer; brain tumor; craniocerebral trauma; severe brain injury; hemorrhagic stroke; ischemic stroke; inflammatory brain disease; neurodegenerative disease; bipolar disorder; organic psychological disorders; known intelligence reduction; epilepsy; depression; schizophrenia; anxiety disorder; obsessive-compulsive disorder; eating disorder; addictive disorder (alcohol); addictive disorder (drugs); addictive disorder (medication); PTSD; adult ADHD; Personality disorder; other (please specify); none of the stated |
|  | Stomachaches in the past three months | Never; less than once per month; once per month; two or three times per month; once per week; more than once per week; every day |
|  | Having felt sick in the past three months | Always; usually; sometimes; occasionally; never |
|  | Having had soft/watery stool during the past three months | No; yes, one day; yes, several days; yes, almost every day |
|  | Having had hard stool/constipation during the past three months | No; yes, one day; yes, several days; yes, almost every day |
|  | Having had an appendicitis |  |
|  | Having relatives suffering from an inflammatory intestinal disease | No; yes, Crohn’s disease; yes, ulcerative colitis; yes, appendicitis; I don’t know |
|  | Being a vegetarian or vegan |  |
|  | Frequency of meat consummation | Never; daily (number of meals); weekly (number of meals) monthly (number of meals); less than monthly; I don’t know |
|  | Frequency of fish consummation | Never; daily (number of meals); weekly (number of meals) monthly (number of meals); less than monthly; I don’t know |
|  | Frequency of fruit consummation | Never; daily (number of pieces); weekly (number of pieces) monthly (number of pieces); less than monthly; I don’t know |
|  | Frequency of vegetable consummation | Never; daily (number of pieces); weekly (number of pieces) monthly (number of pieces); less than monthly; I don’t know |
|  | Frequency of fibre consummation (beans, peas, cabbage, broccoli, …) | Never; daily (number of pieces); weekly (number of pieces) monthly (number of pieces); less than monthly; I don’t know |
|  | Frequency of consuming sugar-containing drinks | Never; daily (number of glasses); weekly (number of glasses) monthly (number of glasses); less than monthly; I don’t know |
|  | Amount of chocolate eaten per month | None; one bar of 200g; 2-5 bars of 200g; 6-10 bars of 200g; more |
|  | Type of chocolate | Dark; milk; white |
|  | Amount of milk consumed | None; less than 1 glass per day; 1-2 glasses per day; 3-4 glasses per day; more |
|  | Type of consumed milk | Long-life; pasteurized |
|  | Having ever been infected with COVID-19 |  |

**Table S1.** Overview of assessed covariates related to sociodemographics, health, and habits.

# Ambulatory Assessments

## EMA beep schedule

| **Day 1** | **Day 2** | **Day 3** | **Day 4** | **Day 5** | **Day 6** |
| --- | --- | --- | --- | --- | --- |
| 8:02 | 7:41 | 7:57 | 8:00 | 7:31 | 7:54 |
| 9:02 | 9:32 | 9:05 | 9:17 | 9:33 | 10:03 |
| 11:12 | 11:12 | 11:24 | 11:27 | 10:32 | 10:41 |
| 13:26 | 13:04 | 12:28 | 12:59 | 12:33 | 12:46 |
| 13:53 | 13:47 | 13:52 | 13:32 | 14:34 | 13:56 |
| 16:21 | 16:28 | 16:07 | 15:08 | 15:12 | 15:12 |
| 17:21 | 17:06 | 16:33 | 17:16 | 17:37 | 16:32 |
| 18:13 | 18:54 | 19:24 | 18:15 | 18:56 | 18:56 |
| 20:17 | 20:49 | 20:11 | 20:00 | 20:25 | 20:14 |
| 21:05 | 22:29 | 21:59 | 22:09 | 22:20 | 22:21 |

**Table S2.** Times of EMA questionnaires during the EMA weeks for all participants

## EMA questionnaire


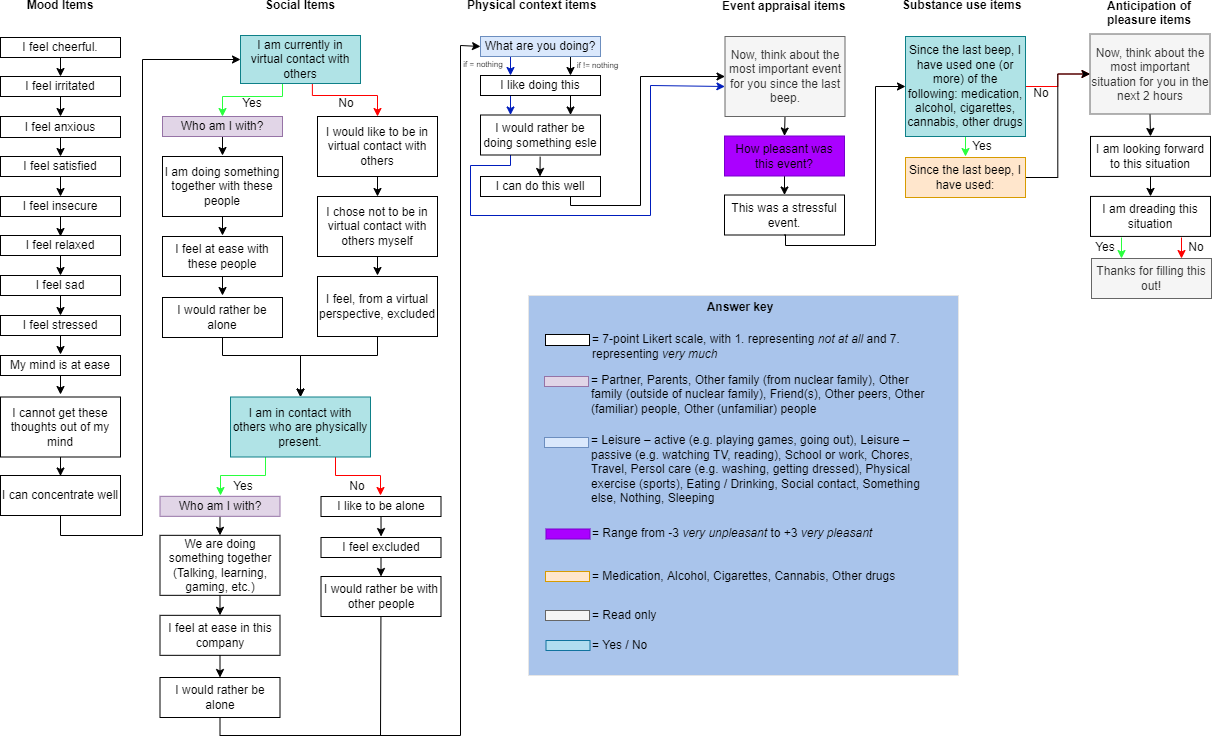


**Figure S8. EMA general questionnaire.** When participants opened the app in the morning, the questionnaire additionally included the items “At what time did you fall asleep?”, “At what time did you wake up?”, “I slept well”, and “I feel rested”.
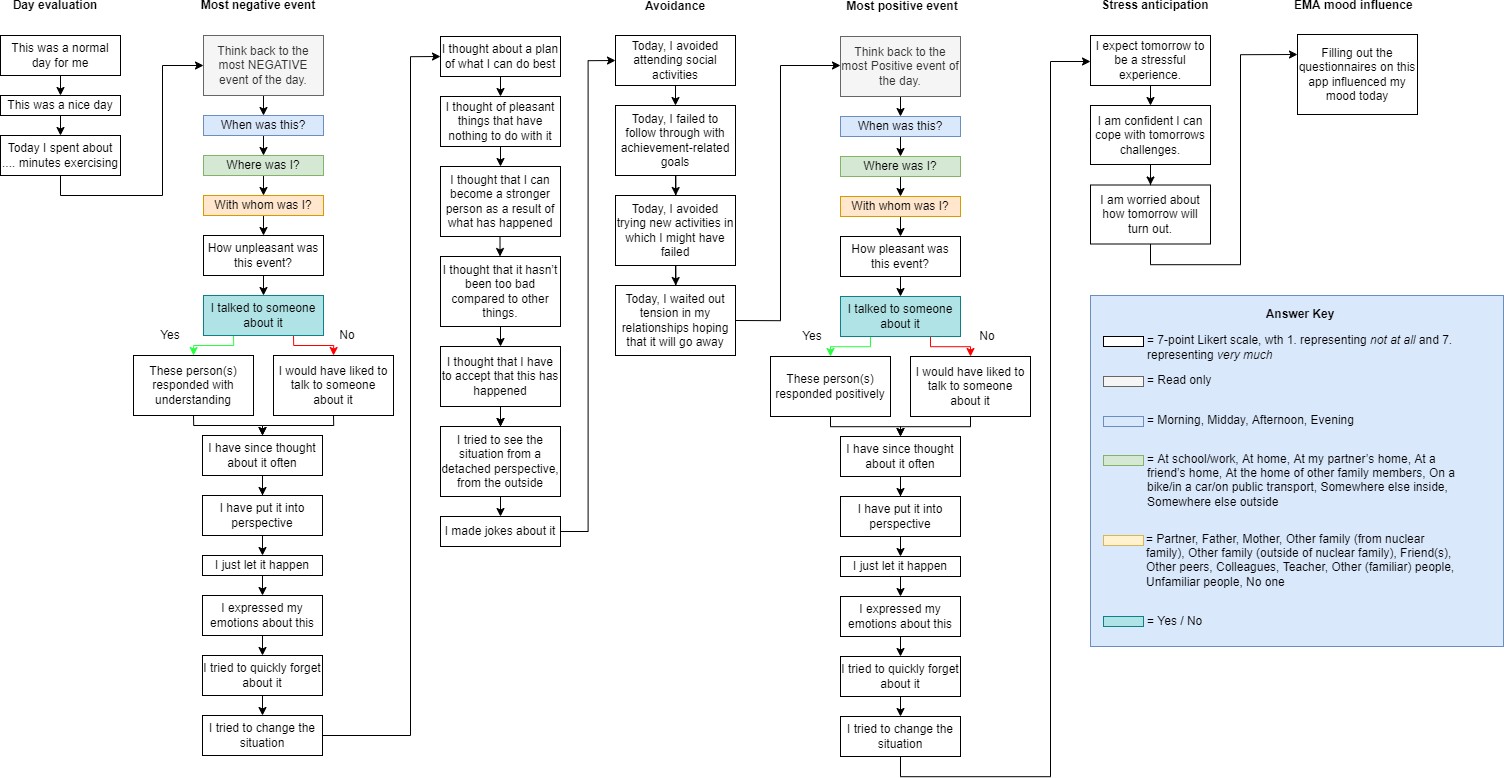


#### Figure S9. EMA evening questionnaire.

# Remuneration schedule

|  | **Baseline** | | **M1** | | | | **M2** | | | | **M3** | | | | **M4** | | | | **M5** | | | | **M6** | | | | **M7** | | | | **M8** | | | | **M9** | | | |
| --- | --- | --- | --- | --- | --- | --- | --- | --- | --- | --- | --- | --- | --- | --- | --- | --- | --- | --- | --- | --- | --- | --- | --- | --- | --- | --- | --- | --- | --- | --- | --- | --- | --- | --- | --- | --- | --- | --- |
|  | **d1** | **d2** | **w1** | **w2** | **w3** | **w4** | **w1** | **w2** | **w3** | **w4** | **w1** | **w2** | **w3** | **w4** | **w1** | **w2** | **w3** | **w4** | **w1** | **w2** | **w3** | **w4** | **w1** | **w2** | **w3** | **w4** | **w1** | **w2** | **w3** | **w4** | **w1** | **w2** | **w3** | **w4** | **w1** | **w2** | **w3** | **w4** |
|  |  | |  | | | |  | | | |  | | | |  | | | |  | | | |  | | | |  | | | |  | | | |  | | | |
| EUR |  | 50 + MID win |  |  |  |  |  |  |  |  |  |  |  | 60 |  |  |  |  |  |  |  |  |  |  | 120 + lottery |  |  |  |  |  |  |  |  |  |  |  | 60 |  |
| NIS |  | 350 + MID win |  |  |  |  |  |  |  |  |  |  |  | 240 |  |  |  |  |  |  |  |  |  |  | 480 + lottery |  |  |  |  |  |  |  |  |  |  |  | 104 |  |
| PLN |  | 400 + MID win |  |  |  |  |  |  |  |  |  |  |  | 200 |  |  |  |  |  |  |  |  |  |  | 400 + lottery |  |  |  |  |  |  |  |  |  |  |  | 200 |  |

**Table S3. Remuneration schedule.** Participants received at least 290 EUR / 1224 NIS / 1200 PLN for complete study participation in total. Further, they received about 10€ on average during the reward task (monetary incentive delay task, MID), and were included in a lottery to win one out of 3 times 100 EUR / 400 NIS / 400 PLN per site if they accomplished all assessments until (and including) month six, week three.
