# Supplementary material for: Dynamic Modelling of Mental Resilience in Young Adults: Protocol for a Longitudinal Observational Study (DynaM-OBS)
Source: JMIR Res Protoc. 2023 Jul 4;12:e39817. doi: 10.2196/39817 (PMC10354714; doi:10.2196/39817)
Supplement: Multimedia Appendix 4 [file resprot_v12i1e39817_app4.docx]

This is a Multimedia Appendix to a full manuscript published in the J Med Internet Res. For full copyright and citation information see http://dx.doi.org/10.2196/jmir.39817

|  |  |  | |  | **Baseline** | | **Phase I** | | | | | | | | | | | | | | | | | | | | | | | | | **Phase II** | | | | | | | | | | | |
| --- | --- | --- | --- | --- | --- | --- | --- | --- | --- | --- | --- | --- | --- | --- | --- | --- | --- | --- | --- | --- | --- | --- | --- | --- | --- | --- | --- | --- | --- | --- | --- | --- | --- | --- | --- | --- | --- | --- | --- | --- | --- | --- | --- |
|  |  |  | | **Scree-ning** |  | | **M1** | | | | **M2** | | | | **M3** | | | | **M4** | | | | **M5** | | | | | **M6** | | | | **M7** | | | | **M8** | | | | **M9** | | | |
|  |  |  |  |  | **d1** | **d2** | **w1** | **w2** | **w3** | **w4** | **w1** | **w2** | **w3** | **w4** | **w1** | **w2** | **w3** | **w4** | **w1** | **w2** | **w3** | **w4** | | **w1** | **w2** | **w3** | **w4** | **w1** | **w2** | **w3** | **w4** | **w1** | **w2** | **w3** | **w4** | **w1** | **w2** | **w3** | **w4** | **w1** | **w2** | **w3** | **w4** |
| **Inclusion** |  | Inclusion criteria | | **x** |  |  |  |  |  |  |  |  |  |  |  |  |  |  |  |  |  |  | |  |  |  |  |  |  |  |  |  |  |  |  |  |  |  |  |  |  |  |  |
|  |  | MINI Interview | |  | **x** |  |  |  |  |  |  |  |  |  |  |  |  |  |  |  |  |  | |  |  |  |  |  |  |  |  |  |  |  |  |  |  |  |  |  |  |  |  |
|  |  | Drug screening | |  | **x** | **x** |  |  |  |  |  |  |  |  |  |  |  |  |  |  |  |  | |  |  |  |  |  |  |  |  |  |  |  |  |  |  |  |  |  |  |  |  |
|  | **Online Questionnaires** | | |  |  |  |  |  |  |  |  |  |  |  |  |  |  |  |  |  |  |  | |  |  |  |  |  |  |  |  |  |  |  |  |  |  |  |  |  |  |  |  |
| **Stressor Reactivity** |  | GHQ-28 | General Health Questionnaire | **x** |  |  | **x** |  | **x** |  | **x** |  | **x** |  | **x** |  | **x** |  | **x** |  | **x** |  | | **x** |  | **x** |  | **x** |  | **x** |  |  |  | **x** |  |  |  | **x** |  |  |  | **x** |  |
|  |  | MIMIS | Mainz Inventory of Microstressors |  |  |  | **x** |  | **x** |  | **x** |  | **x** |  | **x** |  | **x** |  | **x** |  | **x** |  | | **x** |  | **x** |  | **x** |  | **x** |  |  |  | **x** |  |  |  | **x** |  |  |  | **x** |  |
|  |  | COV Stress | Covid-related Stressors |  |  |  | **x** |  | **x** |  |  |  | **x** |  |  |  | **x** |  |  |  | **x** |  | |  |  | **x** |  |  |  | **x** |  |  |  | **x** |  |  |  | **x** |  |  |  | **x** |  |
|  |  | LEQ | Life Event Questionnaire | **x** |  |  | **x** |  |  |  |  |  |  |  |  |  | **x** |  |  |  |  |  | |  |  |  |  |  |  | **x** |  |  |  |  |  |  |  |  |  |  |  | **x** |  |
|  |  | SCL-90-R | Symptom Checklist 90 Revised |  |  |  | **x** |  |  |  |  |  |  |  |  |  | **x** |  |  |  |  |  | |  |  |  |  |  |  | **x** |  |  |  |  |  |  |  |  |  |  |  | **x** |  |
|  |  | WHO-DAS | WHO Disability Assessment Scale |  |  |  | **x** |  |  |  |  |  |  |  |  |  | **x** |  |  |  |  |  | |  |  |  |  |  |  | **x** |  |  |  |  |  |  |  |  |  |  |  | **x** |  |
| **Potential Resilience and Risk Factors** | **Neuropsychological battery** | | |  | **x** |  |  |  |  |  |  |  |  |  |  |  |  |  |  |  |  |  | |  |  |  |  |  |  |  |  |  |  |  |  |  |  |  |  |  |  |  |  |
|  | **Neuroimaging battery** | | |  |  | **x** |  |  |  |  |  |  |  |  |  |  |  |  |  |  |  |  | |  |  |  |  |  |  |  |  |  |  |  |  |  |  |  |  |  |  |  |  |
|  | **Online Questionnaires** | | |  |  |  |  |  |  |  |  |  |  |  |  |  |  |  |  |  |  |  | |  |  |  |  |  |  |  |  |  |  |  |  |  |  |  |  |  |  |  |  |
|  |  | PASSp | Positive Appraisal Style Scale – process-based |  |  |  | **x** |  | **x** |  | **x** |  | **x** |  | **x** |  | **x** |  | **x** |  | **x** |  | | **x** |  | **x** |  | **x** |  | **x** |  |  |  | **x** |  |  |  | **x** |  |  |  | **x** |  |
|  |  | Other primary resilience and risk factors | |  |  |  | **x** |  |  |  |  |  |  |  |  |  |  |  |  |  |  |  | |  |  |  |  |  |  |  |  |  |  |  |  |  |  |  |  |  |  | **x** |  |
|  |  | Secondary resilience and risk factors | |  |  |  | **x** |  |  |  |  |  |  |  |  |  |  |  |  |  |  |  | |  |  |  |  |  |  |  |  |  |  |  |  |  |  |  |  |  |  |  |  |
|  |  | Sports and mental activities | |  |  |  |  |  |  |  |  |  |  |  |  |  | **x** |  |  |  |  |  | |  |  |  |  |  |  | **x** |  |  |  |  |  |  |  |  |  |  |  | **x** |  |
|  | **Bio-samples** | | |  |  |  |  |  |  |  |  |  |  |  |  |  |  |  |  |  |  |  | |  |  |  |  |  |  |  |  |  |  |  |  |  |  |  |  |  |  |  |  |
|  |  | EDTA Blood | DNA/DNA-methylation |  | **x** |  |  |  |  |  |  |  |  |  |  |  |  |  |  |  |  |  | |  |  |  |  |  |  | **x** |  |  |  |  |  |  |  |  |  |  |  |  |  |
|  |  | Stool | Microbiome |  |  |  | **x** |  |  |  |  |  |  |  |  |  | **x** |  |  |  |  |  | |  |  |  |  |  |  | **x** |  |  |  |  |  |  |  |  |  |  |  |  |  |
|  |  | Saliva | Cortisol during MRI assessment |  |  | **x** |  |  |  |  |  |  |  |  |  |  |  |  |  |  |  |  | |  |  |  |  |  |  |  |  |  |  |  |  |  |  |  |  |  |  |  |  |
| **Dynamics** | **Ambulatory Assessments** | | |  |  |  |  |  |  |  |  |  |  |  |  |  |  |  |  |  |  |  | |  |  |  |  |  |  |  |  |  |  |  |  |  |  |  |  |  |  |  |  |
|  |  | EMA | Ecological Momentary Assessment |  |  |  |  | **x** |  |  |  | **x** |  |  |  | **x** |  |  |  | **x** |  |  | |  | **x** |  |  |  | **x** |  |  |  |  |  |  |  |  |  |  |  |  |  |  |
|  |  | EPA | Ecological Physiological Assessment |  |  |  |  | **x** |  |  |  | **x** |  |  |  | **x** |  |  |  | **x** |  |  | |  | **x** |  |  |  | **x** |  |  |  |  |  |  |  |  |  |  |  |  |  |  |

**Multimedia Appendix 4.** Overview of the measures used and the days (d), weeks (w) and months (M) from baseline, at which they are assessed (x). Resilience and risk factors (RFs) are grouped into primary and secondary RFs. Primary RFs are of main interest in the current study based on previous findings and theoretical background of our consortium [9, 36, 40], while secondary RFs are based on hypotheses drawn from the literature. The complete lists of inventories used to assess primary and secondary RFs are provided in the main text.
